# The effects of acute and chronic exercise on immune markers of TH1/TH2 cells in older adults: a systematic review

**DOI:** 10.3389/fphys.2025.1453747

**Published:** 2025-02-11

**Authors:** Thiago Henrique Teodoro, Katerine Palharini Manfrin Costa, Jonato Prestes, José Campanholi, James Navalta, Guilherme Borges Pereira

**Affiliations:** ^1^ Laboratory of Clinical Exercise Physiology, Department of Physiological Sciences, Federal University of São Carlos, São Carlos, Brazil; ^2^ Graduation Program on Physical Education, Catholic University of Brasilia, Brasília, Brazil; ^3^ Department of Morphology and Pathology, Federal University of São Carlos, São Carlos, Brazil; ^4^ Department of Kinesiology and Nutrition Sciences, University of Nevada, Las Vegas, Las Vegas, NV, United States

**Keywords:** acute exercise, chronic exercise, immune system, exercise immunology, Th1 cells, Th2 cells, immunosenescence, humans

## Abstract

**Purpose:**

Imbalance between Th1 and Th2 cells correlated with increased disease incidence, is well-documented in the older adult. Both acute and chronic exercise induce a transient shift in organic homeostasis, modulating the immune system and impacting the balance between Th1 and Th2 cells. This review investigates the impact of acute and chronic exercise on immune markers of Th1 and Th2 cells in the older adults.

**Methods:**

This study was conducted as a systematic review, following PRISMA guidelines, databases including PubMed, Web of Science, Embase, Science Direct, and Scopus were searched until March 2024, identifying randomized controlled trials and prospective observational studies that examined the effects of acute and chronic exercise on intracellular and surface markers, cytokines, and immunoglobulins in older adults. Studies involving animal subjects, isolated cells, diseased patients, or exposure to medications and drugs were excluded. The quality of the selected studies was assessed using the Cochrane risk of bias tool (ROB2), with data organized and presented in tables and figures.

**Results:**

Fourteen studies with 525 participants were included in the analysis. An acute session significantly increased serum IL-4, IL-6, and IL-10 levels immediately afterward, returning to baseline within 1 hour at moderate to high intensities. Chronic exercise at moderate to high intensities reduced serum TNF-α, IL-6, and the CD4/CD8 ratio, while increasing IL-10 levels after 24 weeks. Intracellular, other surface markers and cytokines, and immunoglobulins were not analyzed.

**Conclusion:**

Chronic exercise decreases serum TNF-α and IL-6 levels, lowers the CD4/CD8 ratio, and increases IL-10 after 24 weeks, aiding Th1 and Th2 balance. Acute exercise temporarily increases serum IL-4, IL-6, and IL-10 levels, returning to baseline within an hour, indicating short-term immune modulation of Th1/Th2 balance.

**Systematic Review Registration:**

https://www.crd.york.ac.uk/prospero/display_record.php?ID=CRD42021244426, Identifier CRD42021244426.

## 1 Introduction

Physical exercise is recognized as a vital strategy for enhancing overall health, especially in relation to aging. Benefits of exercise include increased cardiorespiratory fitness, reduced blood pressure, improved glycemic control, and enhanced bone density and muscle function. Additionally, it significantly improves sleep quality, mental health, and overall life quality. Furthermore, exercise stimulates the release of myokines—cytokines and peptides produced by skeletal muscle—which play key roles in mediating metabolic and immune responses, contributing to these health benefits (Costa Rosa and Vaisberg, 2002; Terra et al., 2012; Leandro et al., 2007; Sellami et al., 2021; Supriya et al., 2021; Oliveira et al., 2021; Silva et al., 2022; Del Rosso et al., 2023; Papagianni et al., 2023; Khalafi et al., 2024; Luo et al., 2024).

However, aging also poses complex challenges to the immune system, such as the dysregulation of Th1 and Th2 cellular balance, leading to immunosenescence. This immunological shift heightens susceptibility to chronic degenerative diseases, autoimmune disorders, infections, and diminishes vaccine efficacy (Timmerman et al., 2008; Agondi et al., 2012; Raynor et al., 2012; Fulop et al., 2018; Papp et al., 2021; Salimans et al., 2022). Exercise training has been shown to mitigate these effects by restoring Th1 and Th2 balance, enhancing immune function in older adults (Luo et al., 2024; Khalafi et al., 2023).

Recent studies highlight the positive impact of exercise on immune responses in the older adults. The release of myokines, such as interleukin-6 (IL-6) during muscle contractions, not only influences metabolic pathways but also modulates immune function, reinforcing the systemic benefits of physical activity. A systematic review and meta-analysis of randomized controlled trials involving 539 participants aged 60 or older reported that resistance training effectively reduced inflammatory markers such as C-reactive protein (CRP), tumor necrosis factor-alpha (TNF-α), interleukin-10 (IL-10), and potentially interleukin-6 (IL-6) (Kim and Yeun, 2022). Additionally, a systematic review by Barni and coauthors (Barni et al., 2023) demonstrated that physical training alongside the COVID-19 vaccine boosts serum antibody levels, reinforcing earlier findings on the beneficial effects of exercise on vaccine responses (Bohn-Goldbaum et al., 2022). Regular physical exercise potentially enhances immune defense and reduces susceptibility to pathogens, including viruses, by modulating the balance of Th1, Th2, regulatory T cells (Treg), and T helper 17 (Th17) cells. However, acute, high-intensity, or prolonged exercise can lead to transient immunosuppression, known as the “open window,” associated with an increased risk of opportunistic infections, particularly respiratory viral infections (Nieman et al., 1989; Nieman, 1994; Shephard and SHEK, 1996; Matthews et al., 2002).

Naive CD4^+^ T cells, upon activation, can differentiate into various subsets, including type 1 (Th1), type 2 (Th2), Th9, regulatory T cells (Treg), Th17, T follicular helper cells (Tfh), and Th22 lymphocytes, depending on the cytokine environment and other signaling factors. Th1 cells are activated in the presence of IL-12 and Interferon-gamma (IFN-γ), characterized by transcription factors like T-bet and signal transducer and activator of transcription 4 (STAT4), producing cytokines such as IL-2, IFN-γ, lymphotoxin-α, and tumor necrosis factor-beta (TNF-β). These cytokines activate macrophage bactericidal activity and induce B cells to facilitate phagocytosis through opsonin binding like immunoglobulin G (IgG) or complement fragments (Zhou et al., 2012). Conversely, Th2 cells, important for humoral immunity, differentiate into cells secreting IL-4, IL-5, IL-10, and IL-13, mediated by transcription factors GATA-3 and STAT6. IL-5 activates immune reactions against parasites, and IL-4 induces Ig class switching in B lymphocytes, proving effective against extracellular pathogens (Stark et al., 2019).

Despite extensive discussions on leukocytes, cytokine concentrations, and physical exercise in the literature (Supriya et al., 2021; Del Rosso et al., 2023; Khalafi et al., 2024; Luo et al., 2024; Salimans et al., 2022; Kim and Yeun, 2022; Gleeson, 1985; Campbell and Turner, 2018; Ringleb et al., 2024; Page et al., 2021) a review explicitly addressing the impact of acute and chronic physical exercise on markers of Th1 and Th2 cells in older adults is lacking.

The Th1/Th2 balance plays a critical role not only in antigen-specific immune responses but also in broader physiological and pathological processes. An imbalance in this axis has been linked to chronic inflammatory states, increased susceptibility to autoimmune diseases, and reduced effectiveness of immune surveillance mechanisms, particularly in aging populations (Fulop et al., 2018; Papp et al., 2021). These systemic consequences of Th1/Th2 imbalance are pivotal to understanding the progression of immunosenescence and its clinical implications, such as heightened vulnerability to infections and chronic degenerative diseases (Agondi et al., 2012; Raynor et al., 2012). Our focus on this aspect is driven by its central role in immune homeostasis and the potential of exercise to modulate these pathways, thereby mitigating the negative effects of aging on the immune system (Papp et al., 2021; Campbell and Turner, 2018).

The addition of this knowledge will be crucial for understanding whether, after a physical exercise session, there is a period of immunosuppression due to reduced immune competence (decrease in immune activity and/or efficiency) and whether this occurrence can indeed characterize the “open window” period, consequently increasing the risk of opportunistic infections. Furthermore, it will explore whether chronic and regular exercise training has a beneficial effect on the immune system and whether, due to leukocyte polarization toward a specific Th subgroup, it can promote bio-positive changes in the Th1/Th2 cell balance.

Therefore, this systematic review aimed to elucidate the acute and chronic effects of exercise on intracellular and surface markers (cluster of differentiation), secreted factors (cytokines), and immunoglobulins (Ig) of Th1 and Th2 cells in studies conducted specifically with older adults.

## 2 Materials and methods

The current systematic review has been conducted in accordance with the reporting guidance provided in the Preferred Reporting Items for Systematic Reviews and Meta-Analyses (PRISMA) checklist, flow diagram and Explanation and Elaboration document (Ouzzani et al., 2016) and the Cochrane Handbook for Systematic Reviews of Interventions Version 6.3, 2021. The protocol was registered in the Prospective Registry of Systematic Reviews (PROSPERO) with ID: CRD42021244426.

### 2.1 Search strategy

A comprehensive literature search was performed using five electronic databases including PubMed, EMBASE, Science Direct, Web of Science, and Scopus. We included studies published up until March 2024, with no limitations regarding the initial date of publication, and any discrepancies during the selection process were resolved through consensus among the reviewers K.P.M.C., T.H.T., and G.B.P.

The search strategy was performed using the Boolean operators “AND” and “OR”, and a combination of the derived equivalent MESH term key words, see details Supplementary File S1 Additional filters included English or Portuguese languages, human participants, and article/document type when they were available in electronic databases.

We focused on a selection of observational and experimental designs, including prospective cohort studies and randomized controlled trials (RCTs), with an emphasis on cluster RCTs and self-controlled designs where participants acted as their own controls.

### 2.2 Study eligibility and selection

Studies were eligible for inclusion if they met the following PICO (population, intervention, comparison, and outcome) criteria (Costa Rosa and Vaisberg, 2002): For population, human participants with a mean age ≥60 years, regardless of sex or fitness status, were included. Participants were classified into subgroups based on their training status (untrained or trained). For the untrained subgroup, studies recruited participants who had no history of regular exercise training for at least 6 months prior to the study. For the trained and athlete subgroup, studies recruited participants who reported at least five scheduled exercise sessions per week for at least 1 year (Terra et al., 2012). For the intervention, studies with any mode of exercise (acute or chronic), irrespective of type (aerobic, resistance, or combined) were included. For acute exercise, studies with a single session of exercise were included. For chronic exercise, studies with exercise duration ≥2 weeks were included. There were no limitations for other exercise characteristics, such as intensity, frequency, and time (Leandro et al., 2007). To investigate the effects of acute exercise, post-exercise values versus pre-exercise values were analyzed. For chronic exercise, the effects of training versus a non-exercise control group were analyzed (Sellami et al., 2021). For outcomes, studies that reported serum or plasma markers of lymphocyte polarization, measured using a fully validated method (Flow Cytometry or ELISA), were included. The detailed outcome measures are listed in Supplementary File S2.

Other inclusion criteria were that articles must have been peer reviewed and published in English. Exclusion criteria were studies with animal models, theses and dissertations, conference abstracts and non-original studies. Chronic exercise studies with non-randomized trials or studies without control groups were excluded.

### 2.3 Data extraction and synthesis

The ‘Rayyan tool’ was used to extract and record information (Ouzzani et al., 2016). Data extracted included (Costa Rosa and Vaisberg, 2002): study characteristics. Including publication year, and study design, allocation of participants and group control (Terra et al., 2012); characteristics of participants including sample size, biological sex, age and training status (Leandro et al., 2007); exercise training characteristics, i.e., mode, duration, intensity, time, and frequency, and (Sellami et al., 2021) data of outcome variable and assessment methods. Data extraction was performed independently by two authors (KPMC and THT), and potential discrepancies between the authors were resolved through consensus with other authors (GBP).

### 2.4 Quality assessment

Study quality was assessed using the ROB2 tool provided by the Cochrane Risk of Bias (De Carvalho et al., 2013; Moseley et al., 2019). The ROB2 tool contains five domains for analyzing the risk of bias, which are (Costa Rosa and Vaisberg, 2002): risk of bias due to the randomization process (Terra et al., 2012); risk of bias due to deviations from intended interventions (Leandro et al., 2007); risk of bias due to missing outcome data (Sellami et al., 2021); risk of bias in outcome measurement (Supriya et al., 2021); risk of bias in selection of the reported result. The tool also allows researchers to classify each domain into three levels: low risk of bias; some concerns (unclear); high risk of bias, allowing for an overall result for each investigated study. This tool has similarities with the analysis method of the Physiotherapy Evidence Database (PEDro) scale, in which both can be used for the same purpose of stratifying the risk of bias (Moseley et al., 2019; Higgins et al., 2011). Two authors (KPMC and THT) assessed the quality of included studies, and any disagreements were resolved via consensus with other authors (GBP).

## 3 Results

### 3.1 Literature search

**FIGURE 1:**
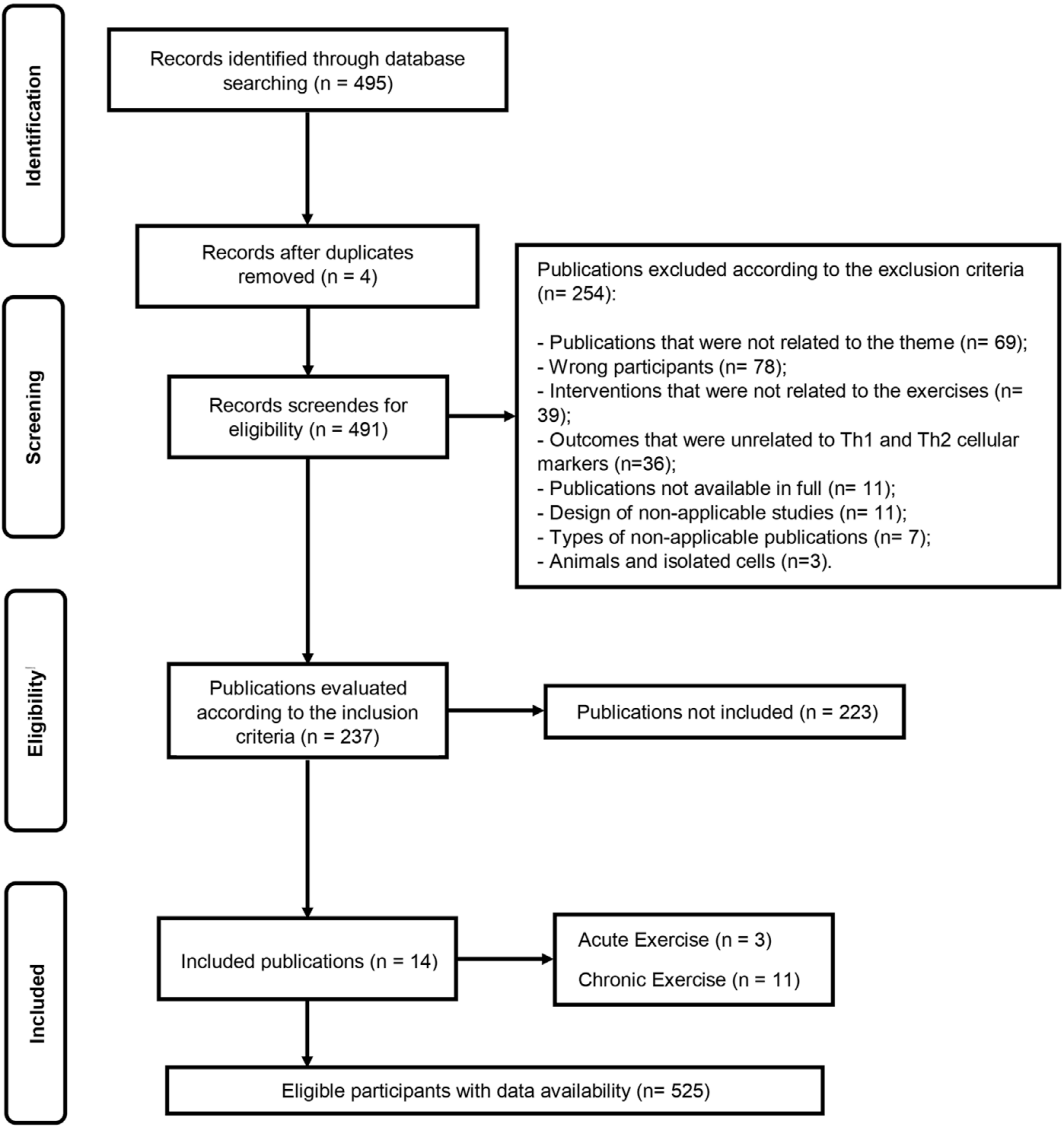
Flow diagram of systematic literature search according to PRISMA.

The initial database search identified 495 records (Figure 1). After duplicate removal, 491 articles remained for screening based on titles and abstracts. Following eligibility screening, 254 studies were excluded according to the exclusion criteria, and 237 studies were assessed according to the inclusion criteria, of which 223 were not included. Finally, the remaining 14 articles were included in this systematic review, encompassing data from 525 eligible participants. Among the included studies, 3 studies (Costa Rosa and Vaisberg, 2002; Terra et al., 2012; Leandro et al., 2007) investigated the acute exercise effect, eleven (Costa Rosa and Vaisberg, 2002; Terra et al., 2012; Leandro et al., 2007; Sellami et al., 2021; Supriya et al., 2021; Oliveira et al., 2021; Silva et al., 2022; Del Rosso et al., 2023; Papagianni et al., 2023; Khalafi et al., 2024; Luo et al., 2024) investigated the chronic exercise effect on several markers of Th1 and Th2 cell polarization (Supplementary File S2).

### 3.2 Study design

In the systematic review encompassing 14 studies, 11 were identified as randomized controlled trials (RCTs) (Shimizu et al., 2011; SoWi-young et al., 2013; Rodriguez-Miguelez et al., 2014; Forti et al., 2016; Sbardelotto et al., 2017; Abd El-Kader and Al-Shreef, 2018; Macêdo Santiago et al., 2018; Windsor et al., 2018; Abd El-Kader and Al-Jiffri, 2019; Abd El-Kader et al., 2019; Sahl et al., 2017). The remaining three studies were non-randomized, employing the exercise group as its own control or utilizing exercise as a comparator (Cornish et al., 2018; Minuzzi et al., 2019; Turner and Brum, 2017).

### 3.3 Comparator group

In studies assessing acute interventions, one study employed the experimental group as its own control (Minuzzi et al., 2019). Another study allocated participants based on age and physical condition (Turner and Brum, 2017), while a single study compared exercise outcomes with those of a control group that remained at rest (without exercise) (Abd El-Kader and Al-Jiffri, 2019). Regarding studies focusing on chronic interventions, five studies included participants who did not engage in exercise as the control group (SoWi-young et al., 2013; Rodriguez-Miguelez et al., 2014; Forti et al., 2016; Windsor et al., 2018; Abd El-Kader et al., 2019). One study designated a control group that performed flexibility and toning exercises (Shimizu et al., 2011). Three studies used exercise as a comparator (Sbardelotto et al., 2017; Macêdo Santiago et al., 2018; Sahl et al., 2017). In another, the experimental group served as its own control (Cornish et al., 2018), and in one study, exercise was compared with a control group that remained at rest (without exercise) (Abd El-Kader and Al-Shreef, 2018).

### 3.4 Participant characteristics

The total number of participants in the studies was 525 older adults, with 190 men, 116 women, and 219 participants whose sex was not identified. Of the 14 included studies, four studies included only men (Abd El-Kader and Al-Shreef, 2018; Cornish et al., 2018; Minuzzi et al., 2019; Turner and Brum, 2017), five included both men and women (SoWi-young et al., 2013; Rodriguez-Miguelez et al., 2014; Forti et al., 2016; Sbardelotto et al., 2017; Abd El-Kader and Al-Jiffri, 2019), one included only women (Windsor et al., 2018), and in four studies, sex was not specified (Shimizu et al., 2011; Macêdo Santiago et al., 2018; Abd El-Kader et al., 2019; Sahl et al., 2017). The age of the participants ranged from 60 to 86 years. In a single study, there was a comparison of three age groups, which included young adults (31.8 ± 3.00 years), middle-aged adults (54.2 ± 5.9 years), and master athletes (53.1 ± 8.8 years) (Turner and Brum, 2017).

### 3.5 Exercise characteristics

Within the scope of the 14 included studies, experimental designs varied and encompassed a wide spectrum of physical exercises and their effects on immunological parameters in the older adult. Woods et al. (1999) (Shimizu et al., 2011) assessed the impact of brisk walking, while Abd El-Kader and Al-Jiffri (Abd El-Kader et al., 2019) explored the effects of walking or running on a treadmill. Cycling on a cycle ergometer was investigated in two studies (Abd El-Kader and Al-Jiffri, 2019; Turner and Brum, 2017), and Sahl and coauthors (Cornish et al., 2018). delved into outdoor cycling. Resistance training was the chosen modality in six studies (SoWi-young et al., 2013; Rodriguez-Miguelez et al., 2014; Forti et al., 2016; Sbardelotto et al., 2017; Windsor et al., 2018; Minuzzi et al., 2019). Additionally, two studies (Macêdo Santiago et al., 2018; Sahl et al., 2017) evaluated the combined effects of walking or running with resistance training. Moreover, Sbardelotto and coauthors (Abd El-Kader and Al-Shreef, 2018) provided a comprehensive analysis of three distinct protocols: combined cycling and resistance training, combined water-based exercise and resistance training, and exclusively cycling exercise. Each study implemented a specific exercise prescription, ranging from moderate to high-intensity sessions, to assess their respective influences on immune and inflammatory markers. Full details of training status, duration and intensity with the mode of exercise are summarized in Tables 1, 2.

**TABLE 1:** Characteristics of the studies and the effects of chronic physical exercise on immune system cells in the older adults.

	Study design			Characterization of participants			Exercise characteristics			Outcomes, measurement times and results		
References/country	Participant allocations	Group control	Type of intervention, frequency and duration	N sample	Training status	Age group (mean ± standard deviation)	Exercise modality	Duration of the session (min or h)	Exercise protocols	Variables	Time of measurements	Results
Abd El-Kader e Al-Jiffri, (2019) Saudi Arabia	R	Participants did not perform exercises	Chronicle (3x/week) (24 weeks)	50 M: NI; F: NI	Untrained	61–67 years old	Walking or running on a treadmill	40 min	Maximum incremental Warmup with 75W. for 3 min.; 25W stages. Every 3 min. until exhaustion Speed between 80 and 85 rpm	TNF-α	24 weeks	↓
										IL-6	24 weeks	↓
										IL-10	24 weeks	↑
So et al. (2013) Korea	R	Participants did not perform exercises	Chronicle (3x/week) (12 weeks)	40 M: 13 F: 27	Untrained	65–82 years old	Red color elastic band	1 h	Red elastic band (low resistance); 2 to 3 sets with 15–25 repetitions.	TNF-α	12 weeks	↔
										IL-6	12 weeks	↔
Woods, et al. (1999) United States	R	Participants performed flexibility and toning exercises	Chronicle (3x/week) (24 weeks)	29 M: NI; F: NI	Untrained	65 ± 0.8 years old	Brisk walk	Sessions of 10–15 min. at the beginning of the program Increase to 40 min. continuous in week 12	Submaximal Incremental Light intensity of 50% VO2max With progression to moderate intensity at 60%–65% VO2max	CD3^+^	24 weeks	↔
										CD4^+^	24 weeks	↔
										CD8^+^	24 weeks	↔
Macêdo Santiago et al. (2018) Brazil	R	Participants did not perform exercises	Chronicle (3x/week) (8 weeks	19 M: 0 F: 19	Untrained	60–70 years old	**ST on machines** Leg press, bench press plantar flexion, knee and elbow extension, back pulley, knee flexion, elbow flexion	50 min	Protocol - Bi-set method Phase 1: Adaptation, 2 sets of 15 reps. submax. for 1 week;	TNF-α	8 weeks	↓
									Phase 2: Tests, 8 exercises, 3x week, 8 to 12 maximum repetitions with an increase of 15% of the initial load, with an execution speed of 2 s in concentric action and 2 s in eccentric action High intensity Pause: 1 min. between series	IL-6	8 weeks	↓
Abd El Kader, et al. (2019) Saudi Arabia	R	Exercise as a comparator	Chronicle (3x/week) (24 weeks)	80 M: NI; F: NI	Untrained	61–66 years old	Walk or run;ST on machines: Bench press, elbow extension, lower back, abdominals, 45° leg press, elbow flexion, chair extension	Cyclical exercises: 40 min ST: NI	Protocol 1 - Maximum Cyclic Incremental First 3 months at 60%–70% of HRmax In the following 3 months at 70%–80% of HRmax Protocol 2 - Moderate/high intensityST 3 sets of 8–12 repetitions of 60%–85% of 1RM; Moderate/high intensity Pause: 1 min. between series	TNF-α	24 weeks	↓
										IL-6	24 weeks	↓
										IL-10	24 weeks	↑
Forti, et al. (2016) Belgium	R	Exercise as a comparator	Chronicle (3x/week) (12 weeks)	56 M: 26 F: 30	Untrained	68 ± 5 years old	**ST o** **n machines** Leg press, knee extension, seated row	NI	Protocol 1: High intensity: 2 sets of 10–15 repetitions at 80% of 1RM Low intensity: 1 set of 80–100 repetitions at 20% of 1RM Protocol 2: Mixed low intensity: 1 set of 60 repetitions at 20% of 1RM., followed by 1 set of 10–20 repetitions at 40% of 1RM Pause: 1 min. between series	IL-6	12 weeks	↔
Abd El-Kader and Al-Shreef, (2018) Saudi Arabia	R	Exercise as a comparator	Chronicle (3x/week) (24 weeks)	60 M: NI F: NI	Untrained	61–66 years old	Walk or run ST on machines Bench press, knee and elbow extension, lower back, abdominals, 45°leg press, elbow flexion	Cyclical exercises: 40 min ST: 40 min.	Protocol 1 - Incremental Protocol 1 - Incremental with moderate/high intensity Cyclical exercises: First 3 months 60%–70% of HRmax In the following 3 months at 70%–80% of HRmax ST: Moderate/high intensity: 3 sets of 8–12 repetitions of 60%–85% of 1RM Pause: 1 min. between series	CD3^+^	24 weeks	↑
										CD4^+^	24 weeks	↑
										CD8^+^	24 weeks	↑
										Ratio CD4/C D8	24 weeks	↓
										TNF-α	24 weeks	↓
										IL-6	24 weeks	↓
										IL-10	24 weeks	↑
Sahl, et al. (2017) Denmark	NR	The experiment al group was the control itself	Chronicle (7x/week) (2 weeks)	6 M: 6 F: 0	Trained	61 ± 4 years old	Street cycling	10 h 31 ± 37 min	Continuous - Moderate intensity at 53% ± 1% of VO2peak	TNF-α	2 weeks	↔
										IL-6	2 weeks	↑
Shimizu, et al. (2011) Japan	R	Participants did not perform exercises	Chronicle (2x/week) (12 weeks)	24 M: 7 F: 17	Untrained	61–79 years old	ST on machines: Knee extension, leg press, hip abduction and adduction ST with body weight	NI	ST: Low intensity: 1st and 2nd weeks - 1 set of 15 repetitions at 20% of 1RM ST: Low intensity: 3rd and 4th weeks - 2 sets of 15 repetitions at 30% 1RM ST: Low intensity: 5th to 12th weeks - 2 sets of 15 repetitions at 40% 1RM.	CD3^+^	12 weeks	↔
										CD4^+^	12 weeks	↔
										CD8^+^	12 weeks	↔
Rodriguez- Miguelez, et al. (2014) Spain	R	Participants did not perform exercises	Chronicle (2x/week) (8 weeks)	26 M: 7 F: 19	Untrained	65–78 years old	ST on machines Leg press, elbow flexion on pulley, peck deck	NI	ST: Moderate intensity at 60% of 1RM 1st week: 3 sets - 8 repetitions; 2nd week: 3 sets - 10 repetitions 3rd week: 3 sets - 12 repetitions ST: Moderate/high intensity at 70% of 1RM 4th week 3 sets - 8 repetitions; 5th week 3 sets - 8 repetitions; 6th week 3 sets - 8 repetitions	TNF-α	8 weeks	↔
										IL-6	8 weeks	↓
										IL-10	8 weeks	↑
Sbardelotto, et al. (2017) Brazil	R	Participants did not perform exercises Exercise as a comparator	Chronicle (3x/week) (8 weeks)	55 M: 55 F: 0	Untrained	60–80 years old	Cyclical exercise; Combined - Cyclic exercise in water + ST Combined - Cyclic exercise on dry land + ST	—	1st mesocycle - Incremental Cyclic Exercise Training Program	IL-6	8 weeks	↓
								29 min	1st microcycle - Weeks 1 and 26 sessions with 5 series of 5 min.; Intensity - 70% of HRmax; 1 min active recovery between sets			
								35 min	2nd microcycle - Weeks 3–59 sessions with 4 series of 8 min.; Intensity - 75% of HRmax; 1 min active recovery between sets	IL-10	8 weeks	↔
								47 min	3rd microcycle - Weeks 6–8 9 sessions with 3 series of 15 min.; Intensity - 80% of HRmax 2 min active recovery. between sets	IL-6	8 weeks	↓
Sbardelotto, et al. (2017) Brazil	R	Participants did not perform exercises Exercise as a comparator	Chronicle (3x/week) (8 weeks)	55 M: 55 F: 0	Untrained	60–80 years old	Cyclical exercise Combined - Cyclic exercise in water + ST Combined - Cyclic exercise on dry land + ST	—	1st mesocycle - Incremental Cyclic Exercise Training Program	IL-10	8 weeks	↔
								36 min	1st microcycle - Weeks 1 and 2 TF - 6 sessions - 3 sets −12 repetitions Intensity - 60% of 1RM 2 min active recovery between sets Cyclical training – 5 min series Intensity - 70% of HRmax; 1 min active recovery between sets			
								45 min	2nd microcycle - Weeks 3–5 TF - 9 sessions - 3 sets −10 repetitions Intensity - 70% of 1RM 2 min active recovery between sets Cyclical training – 5 min series Intensity - 75% of HRmax; 1 min active recovery between sets	IL-6	8 weeks	↓
Sbardelotto, et al. (2017) Brazil	R	Participants did not perform exercises Exercise as a comparator	Chronicle (3x/week) (8 weeks)	55 M: 55 F: 0	Untrained	60–80 years old	Cyclical exercise Combined - Cyclic exercise in water + ST Combined - Cyclic exercise on dry land + ST	68 min	3rd microcycle - Weeks 6–8 TF - 9 sessions - 3 sets - 8 repetitions; Intensity of 80% of 1RM. Active recovery of 3 min between sets Cyclical training – 5 min series Intensity - 80% of HRmax 1 min active recovery between sets	IL-10	8 weeks	↔
								—	3rd mesocycle - Incremental Combined training Cyclic training in water + ST			
								44 min	1st microcycle - Weeks 1 and 2 TF - 6 sessions - 2 sets of 30 s at maximum speed Active recovery of 1 min between sets	IL-6	8 weeks	↓
									Cyclical training – 10 min series Intensity - 70% of HRmax 1 min active recovery between sets			
Sbardelotto, et al. (2017) Brazil	R	Participants did not perform exercises Exercise as a comparator	Chronicle (3x/week) (8 weeks)	55 M: 55 F: 0	Untrained	60–80 years old	Cyclical exercise Combined - Cyclic exercise in water + ST Combined - Cyclic exercise on dry land + ST	49 min	2nd microcycle - Weeks 3–5 TF - 9 sessions - 3 sets of 20 s at maximum speed; Active recovery of 1 min between sets Cyclical training – 15 min series Intensity - 75% of HRmax 1 min active recovery between sets	IL-10	8 weeks	↔
								59 min	3rd microcycle - Weeks 6–8 TF - 9 sessions - 4 sets of 15 s at maximum speed; Active recovery of 1 min between sets	IL-6	8 weeks	↓
									Cyclical training – 20 min series Intensity - 80% of HRmax 1 min active recovery between sets	IL-10	8 weeks	↔

R = randomized; NR, Non-Randomized; NI, not informed; ST, strength training; M = male; F = female; VO2max = Maximum oxygen volume; min. = minutes; T. = time; W = watts; HRmax, Maximum heart rate; ↑ = Increased; ↓ = Decreased; im.a = Immediately after; ↔ = No significant differences between groups or in the times evaluated.

**TABLE 2:** Characteristics of the studies and the effects of acute physical exercise on immune system cells in the older adults.

	Study design			Characterization of participants			Exercise characteristics			Outcomes, measurement times and results		
References	Participant allocations	Group control	Type of intervention, frequency and duration	N sample	Training status	Age group (mean ± standard deviation)	Exercise modality	Duration of the session (min or h)	Exercise protocols	Variables	Time of measurements	Results
Windsor, et al. (2018) Australia	R	Exercise as a comparator Participants did not perform exercises	Acute (1 session)	30 M: 26 F: 4	Trained and Untrained	60–86 years old	Cycle ergometer	24 min	Protocol 1 Continuous -Moderate intensity at 40% of peak power	TNF-α	Im.a	↔
											20 min	
											90 min	
										IL-6	Im.a	↔
									Protocol 2: Interval - 12 sets of 1 min. at 70% of peak power; 1 min active recovery interval. at 10% peak power			
											20 min	
											90 min	
										IL-10	Im.a	↔
											20 min	
											90 min	
Minuzzi, et al. (2019) Brazil	NR	Groups were allocated by age and physical condition	Acute (1 session)	39 M: 39 F: 0	Trained	9 young people (31.8 ± 3.00 years old) 10 middle age (54.2 ± 5.9 years old); 20 master athletes (53.1 ± 8.8 years old)	Cycle ergometer	Young people 18.2 ± 7.4 min Middle age: 10.3 ± 3.5 min.; Master athletes 16.8 ± 5.5 min.	Protocol Maximum incremental - Heating with 75W. for 3 min.; 25W stages. Every 3 min. until exhaustion Speed between 80 and 85 rpm	TNF-α	Im.a	↔
											1 h	↔
										IL-4	im.a	↑
											1 h	↓
										IL-6	im.a	↑
											1 h	↓
											Im.a	↑
										IL-10		
											1 h	↓
Cornish, et al. (2018) Canada	NR	Each individual was his control	Acute (3 sessions)	11 H: 11 F: 0	Untrained	72,3 ± 4.9 years old	ST on machines Bench press, shoulder press, seated row, leg press, knee extension, plantar flexion	NI	Protocol 1: 2 sets of 12 repetitions with 60% of 1-RM	IL-6	im. a 3 h/6 h/24 h/48 h	↔
									Protocol 2: 2 sets of 10 repetitions with 72% of 1-RM Protocol 3 3 sets of 6 repetitions with 80% of 1RM Moderate to high intensity; 1 min breaks. between devices and series			

R = randomized; NR, Non-Randomized; NI, not informed; ST, strength training; M = male; F = female; VO2max = Maximum oxygen volume; min. = minutes; T. = time; W = watts; HRmax, Maximum heart rate; ↑ = Increased; ↓ = Decreased; im.a = Immediately after; ↔ = No significant differences between groups or in the times evaluated.

### 3.6 Effects of chronic exercise on immune markers of Th1 and Th2 polarization

**FIGURE 2:**
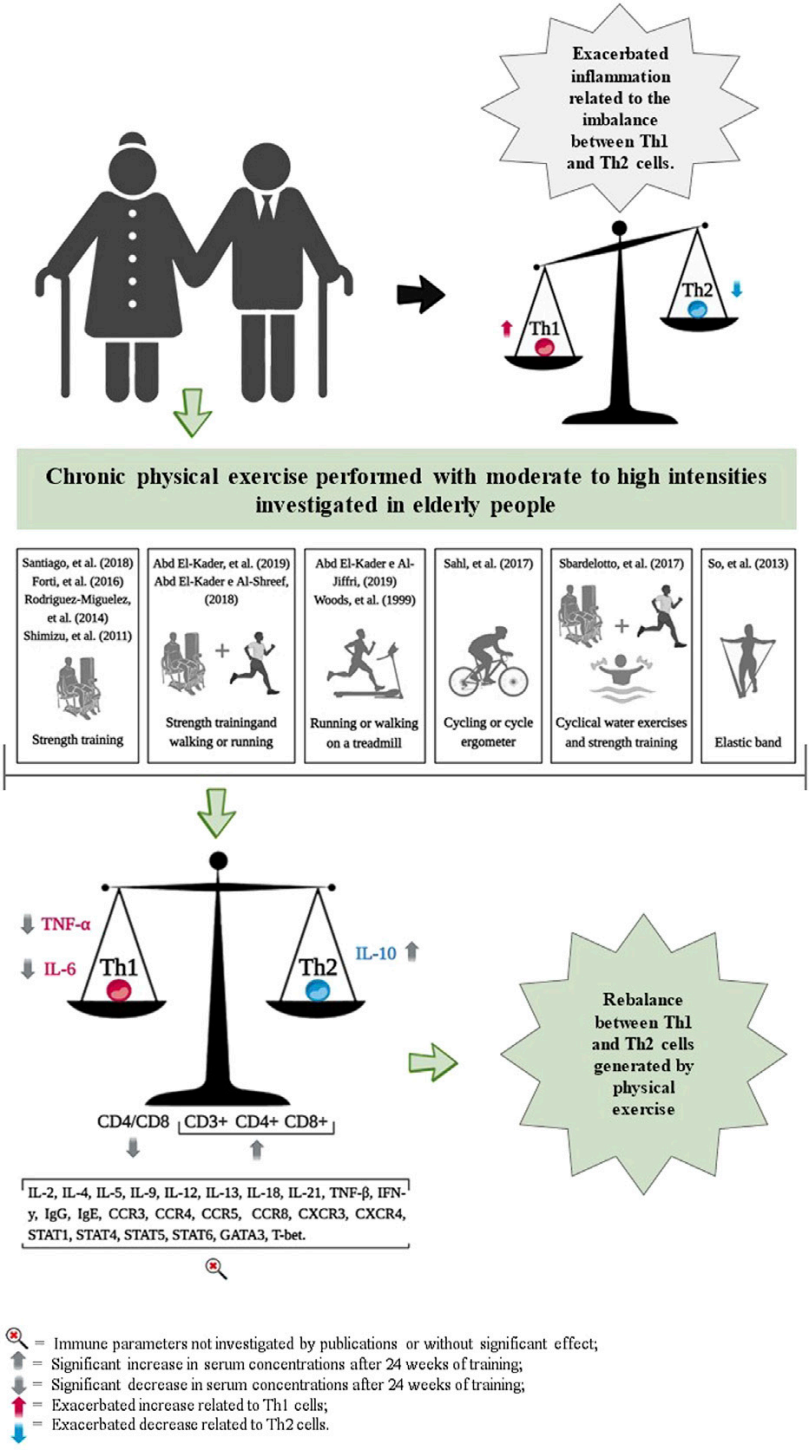
Representation of results related to the impact of chronic physical exercise on immune parameters.

The chronic effects of exercise training on Th1 and Th2 cell markers are presented in Table 1 and Figure 2. The cell surface markers CD3^+^, CD4^+^, CD8^+^, CD4/CD8 ratio, TNF-α, IL-6 and IL-10 were analyzed by selected studies. Woods et al. (1999) (Shimizu et al., 2011) and Shimizu and coauthors (SoWi-young et al., 2013) reported no significant changes in the cell surface markers CD3^+^, CD4^+^, and CD8^+^ following brisk walking exercises at light and moderate intensities, and resistance training at light intensity, over periods of 24 and 12 weeks, respectively. However, Abd El-Kader and Al-Shreef (Macêdo Santiago et al., 2018) demonstrated an increase in the number of CD3^+^, CD4^+^, CD8^+^ cells and a decrease in the CD4/CD8 ratio after 24 weeks of walking or running and resistance training (Table 1).

Furthermore, three studies reported reductions in serum IL-6 concentrations following exercise training, with durations ranging from 8 weeks (Forti et al., 2016; Abd El-Kader and Al-Shreef, 2018; Windsor et al., 2018); and three studies that observed similar reductions over 24 weeks (Macêdo Santiago et al., 2018; Abd El-Kader et al., 2019; Sahl et al., 2017) (Table 1). The study by Sahl and coauthors (Cornish et al., 2018) reported an increase in serum IL-6 concentrations following 2 weeks of strength training. Additionally, So and coauthors (Rodriguez-Miguelez et al., 2014) and Forti and coauthors (Sbardelotto et al., 2017) observed no changes in IL-6 concentrations after 12 weeks of resistance training, which varied from low to high intensities, respectively (Table 1).

The analyses by Abd El-Kader and coauthors (Macêdo Santiago et al., 2018; Sahl et al., 2017) and Rodriguez-Miguelez and coauthors (Forti et al., 2016) demonstrated an increase in serum IL-10 concentrations after 24 weeks of exercise training in older adults. In contrast, studies conducted by Sbardelotto and coauthors (Abd El-Kader and Al-Shreef, 2018) did not show any changes in serum IL-10 concentrations following 8 weeks of training (Table 1).

Reductions in serum TNF-α concentrations were observed after 24 weeks of training (Macêdo Santiago et al., 2018; Windsor et al., 2018; Abd El-Kader et al., 2019; Sahl et al., 2017). However, studies by Rodriguez-Miguelez and coauthors (Forti et al., 2016), Sahl and coauthors (Cornish et al., 2018), and So and coauthors (Rodriguez-Miguelez et al., 2014), reported no changes in TNF-α serum concentrations after 2 weeks, 10 weeks, and 12 weeks of exercise training, respectively (Table 1).

### 3.7 Effects of acute exercise on immune markers of Th1 and Th2 polarization

**FIGURE 3:**
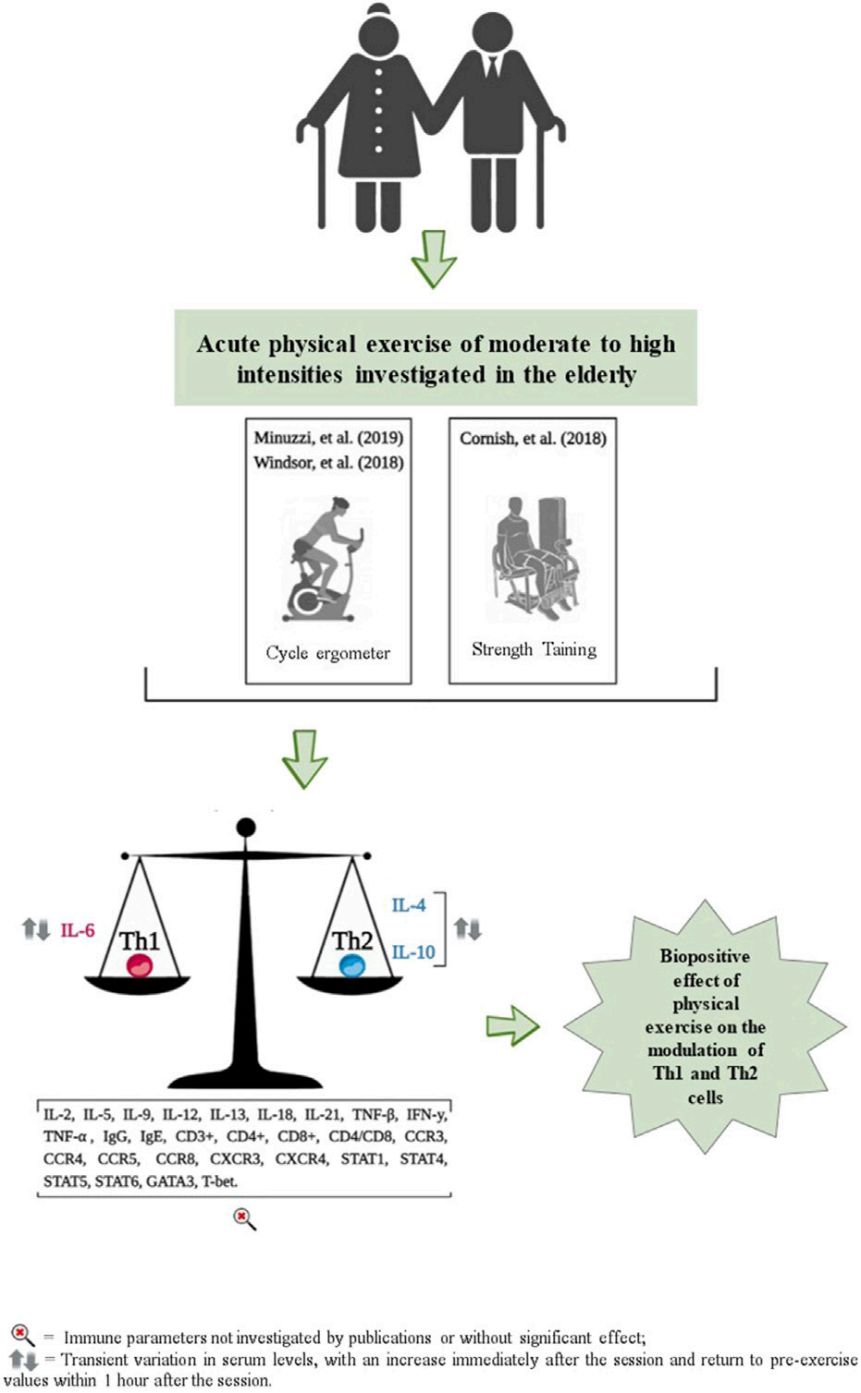
Representation of results related to the impact of acute physical exercise on immune parameters.

The effects of acute exercise on Th1 and Th2 cell markers are detailed in Table 2 and Figure 3. These studies exclusively measured the serum concentrations of TNF-α, IL-4, IL-6, and IL-10. Additionally, they did not investigate the acute effects of exercise on cell surface markers, including CD3^+^, CD4^+^, CD8^+^, and the CD4/CD8 ratio.

No significant changes in serum TNF-α concentrations were observed immediately, or 20 min, 1 h, and 90 min after participants performed cycling exercises (pedaling on a cycle ergometer) at moderate, high, and maximum intensities, as reported by Windsor and coauthors (Abd El-Kader and Al-Jiffri, 2019) and Minuzzi and coauthors (Turner and Brum, 2017), respectively (Table 2). When examining serum IL-4 concentrations, only one study observed an increase immediately after a session of cycling at maximum intensity on a cycle ergometer, followed by a return to pre-exercise values 1 hour later, as reported by Minuzzi and coauthors (Turner and Brum, 2017) (Table 2).

For the serum concentration of IL-6, no significant changes were observed immediately, 20 and 90 min after performing a cycle ergometer session at moderate and high intensities (Abd El-Kader and Al-Jiffri, 2019); and at 6, 24 and 48 h after a strength training session, also at moderate and high intensities (Minuzzi et al., 2019). However, Minuzzi and coauthors (Turner and Brum, 2017) observed a significant increase in IL-6 serum concentration immediately after a maximum intensity cycle ergometer session, which then decreased to pre-exercise levels 1 hour later (Table 2). For serum IL-10 concentrations, Windsor and coauthors (Abd El-Kader and Al-Jiffri, 2019) reported no significant changes immediately, 20 and 90 min after a cycle ergometer session at maximum intensity. However, Minuzzi and coauthors (Turner and Brum, 2017) reported an increase in serum concentrations of IL-10 immediately after, followed by a decrease to pre-values, 1 h after a cycle ergometer session at maximum intensity (Table 2).

### 3.8 Quality assessment and risk of bias evaluation

**FIGURE 4:**
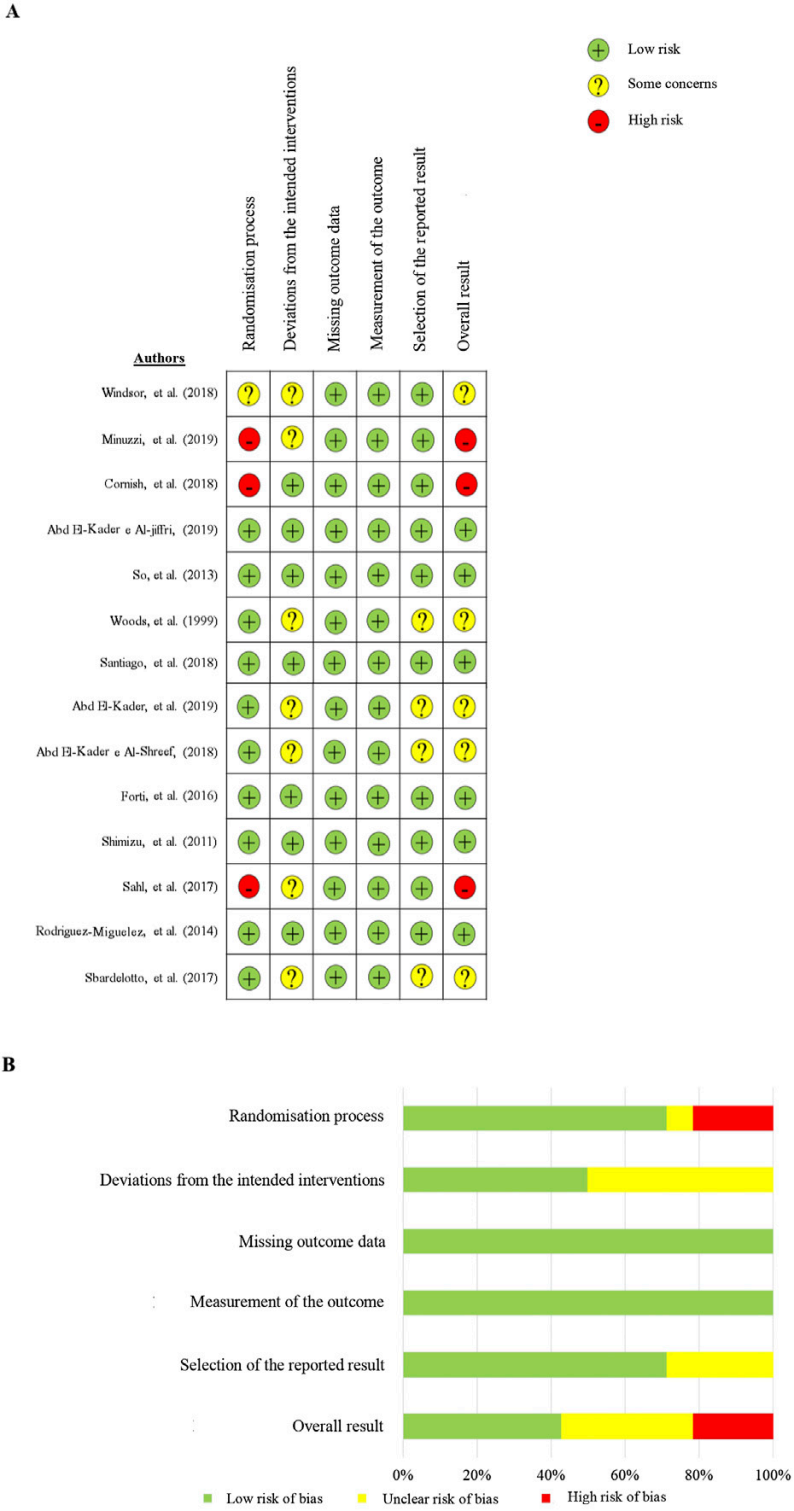
Risk of bias assessment summary, stratified by domains.

After using the Cochrane risk of bias tool, the results are shown in Figure 4 Overall, in three acute studies, the assessments predominantly exhibited a risk of bias with 67% rated as ‘high’ and 33% as having ‘some concerns.’ Among the 11 chronic studies, the evaluations observed 55% with a low risk of bias, 36% with ‘some concerns,’ and 9% with a ‘high’ risk of bias.

## 4 Discussion

Our findings demonstrate that exercise training can modulate the immune response in the older adults. This modulation involves decreasing serum levels of TNF-α and IL-6, reducing the CD4/CD8 ratio, and increasing IL-10 after 24 weeks, thereby contributing to the balance between Th1 and Th2 cells. Additionally, an acute session of exercise also raises serum concentrations of IL-4, IL-6, and IL-10, which return to baseline within an hour post-exercise. These findings suggest that while acute exercise sessions do not disrupt the Th1/Th2 balance, chronic training effects are capable of reducing pro-inflammatory and enhancing anti-inflammatory cytokine levels, thus improving the Th1/Th2 equilibrium. Such chronic adaptations may play a role in mitigating the impact of pathogen infections (e.g., viral infections), assisting in the prevention of various chronic non-communicable diseases, and diminishing the effects of immunosenescence (Supriya et al., 2021; Papagianni et al., 2023; Khalafi et al., 2024; Luo et al., 2024; Papp et al., 2021; Salimans et al., 2022; Page et al., 2021).

Despite the few parameters investigated by the studies selected in this review, the findings indicate that regular physical training can positively modulate the differentiation of effector T lymphocytes and several Th1 and Th2 markers, which can delay or reverse the impact of immunosenescence (Supriya et al., 2021; Wang and Huang, 2005). According to Papp and coauthors (Papp et al., 2021), moderate exercise performed regularly can prevent immunosenescence, reducing the accumulation of T cell clones, promoting their mobilization into the circulation and subsequent extravasation to peripheral tissues, where they will be exposed to apoptosis induced by H2O2, consequently increasing thymic production of naïve T cells.

Only one study showed a decrease in the CD4/CD8 ratio after 24 weeks of moderate to high intensity training, reflecting a greater increase in serum concentrations of CD8 cells compared to CD4 cells (Macêdo Santiago et al., 2018). According to the authors, a decrease in the CD4/CD8 ratio is inherently associated with an increased risk of developing diseases, including cancers, proving to be a strong indicator of high levels of immune activation, replicative senescence and T cell exhaustion (Macêdo Santiago et al., 2018). The proliferation of senescent CD8^+^ T-cell clones, often linked to persistent viral infections, occupies a significant portion of the immune space in older adults. Since the total count of peripheral T-cells is tightly regulated, this overaccumulation of antigen-experienced CD8^+^ T cells can reduce the production of naïve CD4^+^ T cells, limiting the immune system’s ability to respond to new pathogens. Consequently, the CD4/CD8 ratio in sedentary older adults may become excessively elevated, a state that differs from younger populations, where a high CD4/CD8 ratio is generally associated with robust immune health. In older adults, however, such an elevation may reflect impaired immune function and a reduced capacity to mount effective responses against novel pathogens, highlighting the importance of interventions to restore balance. This reduction is naturally facilitated by exercise training, which is thought to improve the Th1/Th2 balance and potentially lower the risk of developing diseases, including cancers and infections. Chronic exercise is hypothesized to trigger apoptosis in apoptosis-resistant and highly differentiated T cells, thereby expanding the T-cell repertoire for new antigens and enhancing overall immune surveillance and reducing infection risk (Terra et al., 2012; Navalta et al., 2007; Nieman, 2000).

The chronic physical training has been shown to have an anti-inflammatory effect in the older adults even when performed at high intensities (Sbardelotto et al., 2017; Abd El-Kader and Al-Shreef, 2018; Macêdo Santiago et al., 2018; Windsor et al., 2018; Abd El-Kader et al., 2019; Sahl et al., 2017) suggesting that it is an efficient countermeasure to prevent or delay the onset of chronic diseases associated with low-grade inflammatory states caused by aging and reduce the incidence of infectious diseases. Several studies indicate that physical inactivity, a pro-inflammatory state, and immunosenescence lead to increased serum levels of TNF-α, IL-6, IL-1, and CRP. These elements are implicated in the development of sarcopenia and a decrease in muscle function, heightening the risk of falls. They also exacerbate morbidity and mortality associated with cardiovascular and metabolic disorders, such as hypertension, obesity, cancer, and type 2 diabetes. Additionally, they amplify susceptibility to and severity of viral infections (Luo et al., 2024; Timmerman et al., 2008; Agondi et al., 2012; Raynor et al., 2012; Fulop et al., 2018; Papp et al., 2021; Sbardelotto et al., 2017; Turner and Brum, 2017; Nieman and Pedersen, 1999).

The scientific literature has traditionally shown that an acute session of cycling, especially if it is vigorous and prolonged, can promote a period called an “open window” of altered immunity and have a detrimental effect on immunological competence, reducing serum levels of immune cells after exercise to below pre-exercise values, increasing the risk of opportunistic infections, mainly respiratory tract infections (Nieman et al., 1989; Matthews et al., 2002; Aoi and Naito, 2019; Nieman and Wentz, 2019; Barardi et al., 2010). However, the findings of this review indicate that a single exercise session (acute response) does not affect the balance of Th1 and Th2 lymphocyte polarization markers, suggesting that it does not characterize an “open window” period of altered immunity. According to Campbell and Turner (Campbell and Turner, 2018), this may occur because an exercise session, instead of suppressing it, is capable of promoting transient changes in the frequency of blood lymphocytes and also promoting an intensified state of regulation and vigilance during and in the hours following exercise (Campbell and Turner, 2018). Together, these data indicate that physical exercise performed at high intensities by older adults does not cause immune suppression, but rather a positive modulation of the function and redistribution of immune system cells.

Despite the insights provided by the studies included in the current review, it was not possible to definitively ascertain the existence of a transient period of immunosuppression. To thoroughly characterize such a state, a comprehensive analysis encompassing a broader range of intracellular markers (e.g., STAT1, STAT4, T-bet, STAT5, STAT6, and GATA-3), cell surface markers (e.g., CD3^+^, CD4^+^, CD8^+^, IFN-γ, CCR5, CXCR3, CD3/CD4 ratio, CD3/CD8 ratio, CD4/CD8 ratio, CCR3, CCR4, CCR8, CXCR4), secreted factors (e.g., TNF-α, TNF-β, IFN-γ, IL-2, IL-12, IL-18, IFN-γ/IL-4 ratio, IL-4, IL-5, IL-6, IL-9, IL-10, IL-13, and IL-21), and immunoglobulins (IgG and IgE) is required. These parameters, which were not explored in the included studies, are crucial for modulating the balance between Th1 and Th2 immune responses (Supriya et al., 2021; Luo et al., 2024; Campbell and Turner, 2018). Although other Th1 and Th2 markers were not investigated, we believe that changes induced by chronic exercise in the distribution of young and memory T cell subtypes, as well as B lymphocytes, indicate a positive regulation of the immune system, restoring their responsiveness (Papp et al., 2021). Physical training can provide an expanded repertoire of young T cells, generated and stimulated by the bone marrow, which migrate to the thymus where thymopoiesis (selection and maturation of T lymphocytes) will occur, allowing these young antigen cells to replace any senescent T cells that underwent apoptosis (Simpson, 2011; Guyton and Hall, 2017; Pedersen and Toft, 2000). This can be explained by the process of lymphocytosis followed by exercise-induced lymphopenia, which stimulates the apoptosis of clone cells and highly differentiated antigen-specific T cells (senescent cells) that are present in large quantities, filling and overloading the immunological space due to the several persistent viral replications that occur throughout life (Guyton and Hall, 2017; Krüger et al., 2008).

However, the lymphopenia process that triggers apoptosis and renewal of the immune space is not limited to the blood compartment alone. It is currently accepted that a mechanism causes the selective recirculation and extravasation of specific subtypes of T lymphocytes from the blood to peripheral tissues and organs after an exercise session. Several pro-apoptotic signals are triggered during exercise, including an increase in glucocorticoids, catecholamines, inflammatory cytokines and reactive oxygen species that are capable of causing changes at the molecular level in the intrinsic and extrinsic pathways that trigger caspases 3, 8 and 9, leading to DNA fragmentation and subsequent apoptosis, preferably of senescent T cells in peripheral tissues and organs. Apoptosis of senescent cells provides a readjustment in the immune repertoire where it will be filled with new, young cells that are stronger and more responsive to new invaders, supposedly as part of an increased immunosurveillance response due to the acute stress generated by exercise (Terra et al., 2012; Campbell and Turner, 2018; Guyton and Hall, 2017; Pereira et al., 2012; Malm, 2006).

However, only one publication presented significant results after a physical exercise session, demonstrating transient variation after the session, indicating that lymphocytosis followed by exercise-induced lymphopenia occurred, mainly at moderate and high intensities, suggesting that a reorganization and rebalancing response occurs between Th1 and Th2 cells instead of generating deregulation and a period of transient immunosuppression after exercise, especially in trained older adults (Guyton and Hall, 2017; Pereira et al., 2012; Malm, 2006; Simpson et al., 2017; Gupta et al., 2018; Wong et al., 2008).

Ample scientific evidence demonstrates that physically active individuals are better protected against viral infections, especially those of the respiratory tract, and when infected, the disease manifests itself in a milder and less aggressive way compared to individuals who do not practice regular physical exercise (Costa Rosa and Vaisberg, 2002; Campbell and Turner, 2018; Warburton and BREDIN, 2017; Ferreira-Júnior et al., 2020). Even so, trained older adults are better able to resist severe physiological and psychological stress, significantly reducing their susceptibility to developing diseases and contracting infections (Khalafi et al., 2023; Guyton and Hall, 2017; Pereira et al., 2012; Malm, 2006; Simpson et al., 2017; Gupta et al., 2018; Wong et al., 2008).

Similar findings were also evidenced in COVID-19, caused by SARS-COV2 infection, where physically active individuals had a lower risk of developing severe symptoms and a lower risk of death compared to physically inactive individuals (Supriya et al., 2021; Guyton and Hall, 2017; Ferreira-Júnior et al., 2020). Interestingly, regular physical exercise modulates the balance between Th1 and Th2 cells, as well as Treg and Th17 cells, which may provide a protective effect against COVID-19 infection, since this disease, like most diseases viral infections, causes an imbalance between these defense cells, especially in vulnerable individuals such as the older adults, obese and/or those with other serious comorbidities (Supriya et al., 2021).

In general, this review portrays the idea that acute or chronic physical exercise performed by older adults at different intensities can stimulate pro- and anti-inflammatory responses related to Th1 and Th2 cells. However, these variations may be transient, with values returning to baseline values after acute exercise, suggesting that there is no “open window” period of altered immunity, even when exercise is performed at high intensities. Chronically, the reduction in serum levels of TNF-α and IL-6, alongside an increase in IL-10 and a lower CD4/CD8 ratio after 24 weeks, reflects a shift toward an anti-inflammatory and Th2-favorable profile, which is essential for maintaining immune homeostasis and reducing systemic inflammation in older adults. Conversely, the acute response to exercise highlights a transient rise in IL-4, IL-6, and IL-10 serum levels, which return to baseline within an hour post-exercise. While IL-4 and IL-10 may transiently contribute to a Th2-favorable environment, the acute elevation of IL-6 likely represents its metabolic role during exercise, such as enhancing glucose uptake and lipid metabolism, rather than a direct effect on the Th1/Th2 axis. These effects are mediated, in part, by myokines, such as IL-6, which are cytokines released by skeletal muscle during contraction that play critical roles in regulating both metabolic and immune functions. Care should be taken when associating the increase of certain molecules (IL-4, IL-6, IL-10, and TNF-α) after a session of physical exercise exclusively with an immune profile, as IL-6, for instance, is primarily released by skeletal muscle contraction and has key metabolic and physiological actions not limited to immune or inflammatory modulation. Together, these findings underscore the dual role of exercise in chronically modulating immune profiles and acutely supporting metabolic and physiological adaptations, reinforcing its systemic benefits for older adults (Khalafi et al., 2024; Luo et al., 2024; Salimans et al., 2022; Campbell and Turner, 2018; Ringleb et al., 2024; Windsor et al., 2018; Pedersen and Toft, 2000).

This review has certain limitations that warrant emphasis. Firstly, a statistical meta-analysis was not conducted to integrate and quantify the findings from the included studies, which could have provided a more accurate assessment of the impact of acute and chronic exercise on Th1 and Th2 markers. Secondly, the selected studies examined a limited range of immunological markers, hindering a comprehensive understanding of how physical exercise affects the Th1/Th2 balance. Thirdly, the inclusion of studies evaluating percentages alongside those assessing absolute numbers introduces variability, thereby limiting the comparability of the findings (Supplementary Tables 1, 2). Finally, chronic studies were shown to have some concerns or a high risk of bias in 45% of cases. We recommend that future research explore the effects of acute and chronic physical exercise on a broader spectrum of markers, including intracellular and cell surface markers, cytokines, and immunoglobulins. Additionally, studies should consider identifying potential pathogenic agents and monitoring signs and symptoms of upper respiratory tract infections in participants following exercise sessions. Furthermore, we encourage the use of best practices to reduce the risk of bias.

## 5 Conclusion

Regular physical training modulates the immune response in the older adults by decreasing TNF-α and IL-6 serum levels, reducing the CD4/CD8 ratio, and increasing IL-10 after 24 weeks, which helps balance Th1 and Th2 cells. An acute exercise session temporarily raises serum IL-4, IL-6, and IL-10 concentrations, returning to baseline within an hour post-exercise, suggesting a transient immune shift with a predominantly metabolic role and minimal impact on Th1/Th2 balance. These adjustments highlight the role of consistent exercise in modulating pro- and anti-inflammatory markers and underscore its significance in supporting immune system health. Further investigation is essential to explore the broader impacts of exercise on Th1 and Th2 cell polarization.

## Data Availability

The datasets presented in this study can be found in online repositories. The names of the repository/repositories and accession number(s) can be found in the article/Supplementary Material.
