# Supplementary material for: The effects of acute and chronic exercise on immune markers of TH1/TH2 cells in older adults: a systematic review
Source: Front Physiol. 2025 Feb 11;16:1453747. doi: 10.3389/fphys.2025.1453747 (PMC11850391; doi:10.3389/fphys.2025.1453747)
Supplement: Supplementary file 1 [file DataSheet2.pdf]

Supplementary file 2

Outcome measures		
	**Th1 cells**	**Th2 cells**
**Intracellular markers**	STAT1, STAT4 e T-bet	STAT5, STAT6 e GATA-3
**Cell surface markers**	CD3+, CD4+, CD8, IFN-y, CCR5, CXCR3 e Razão CD4/CD8	CD3+, CD4+, CD8, CCR3, CCR4, CCR8 e CXCR4
**Secreted factors**	TNF-α, TNF-β, IL-2, IL-12, IL-18 e IFN-y	IL-4, IL-5, IL-6, IL-9, IL-10, IL-13, IL-18 e IL-21
**Immunoglobulin**	IgG	IgE
