# Supplementary material for: The effects of acute and chronic exercise on immune markers of TH1/TH2 cells in older adults: a systematic review
Source: Front Physiol. 2025 Feb 11;16:1453747. doi: 10.3389/fphys.2025.1453747 (PMC11850391; doi:10.3389/fphys.2025.1453747)
Supplement: Supplementary file 2 [file DataSheet1.pdf]

Supplementary file 1

PubMed

https://pubmed.ncbi.nlm.nih.gov/

Database	Descriptors (MESH) and Boolean algebra	Date
Pubmed 1	(((((((exercise[Title]) OR (Exercise Test[Title])) OR (Exercise Therapy[Title])) OR (Physical Endurance[Title])) OR (Sports[Title])) OR (Exercise Tolerance[Title])) OR (training[Title])) AND (th1 cells[Title/Abstract])	March 5, 2024.
Pubmed 2	(((((((exercise[Title]) OR (Exercise Test[Title])) OR (Exercise Therapy[Title])) OR (Physical Endurance[Title])) OR (Sports[Title])) OR (Exercise Tolerance[Title])) OR (training[Title]))) AND (th2 cells[Title/Abstract])	March 5, 2024.
Pubmed 3	(((((((exercise[Title]) OR (physical activity[Title])) OR (training[Title])) OR (exercise therapy[Title])) OR (exercise test[Title])) OR (sports[Title])) OR (exercise tolerance[Title])) AND (Th1-Th2 Balance[Title/Abstract])	March 5, 2024.
Pubmed 4	((((((((exercise[Title]) OR (exercise test[Title])) OR (exercise therapy[Title])) OR (Physical Endurance[Title])) OR (Sports[Title])) OR (Exercise Tolerance[Title])) OR (training[Title])) AND (th1 cells[Title])) OR (th2 cells[Title]) OR (Th1-Th2 Balance[Title])<br><br>Filters: Humans, Adult: 19+ years; Full text, Controlled Clinical Trial, Observational Study, Randomized Controlled Trial, Humans.	March 5, 2024.
Pubmed 5	((((((((exercise[Title]) OR (training[Title])) OR (physical activity[Title])) OR (physical endurance[Title])) OR (exercise test[Title])) OR (exercise therapy[Title])) OR (sports[Title])) OR (exercise tolerance[Title])) AND (CD3+CD4+CD8-[Title/Abstract])	March 5, 2024.
Pubmed 6	((((((((exercise[Title]) OR (training[Title])) OR (physical activity[Title])) OR (physical endurance[Title])) OR (exercise test[Title])) OR (exercise therapy[Title])) OR (sports[Title])) OR (exercise tolerance[Title])) AND (IFN-gamma[Title/Abstract])	March 5, 2024.
Pubmed 7	((((((((exercise[Title]) OR (training[Title])) OR (physical activity[Title])) OR (physical endurance[Title])) OR (exercise test[Title])) OR (exercise therapy[Title])) OR (sports[Title])) OR (exercise tolerance[Title])) AND (Interferon-gamma[Title/Abstract])	March 5, 2024.

Pubmed 8	((((((((exercise[Title]) OR (training[Title])) OR (physical activity[Title])) OR (physical endurance[Title])) OR (exercise test[Title])) OR (exercise therapy[Title])) OR (sports[Title])) OR (exercise tolerance[Title])) AND (IFN-y[Title/Abstract])	March 5, 2024.
Pubmed 9	((((((((exercise[Title]) OR (training[Title])) OR (physical activity[Title])) OR (physical endurance[Title])) OR (exercise test[Title])) OR (exercise therapy[Title])) OR (sports[Title])) OR (exercise tolerance[Title])) AND (IL-18[Title/Abstract])	March 5, 2024.
Pubmed 10	((((((((exercise[Title]) OR (training[Title])) OR (physical activity[Title])) OR (physical endurance[Title])) OR (exercise test[Title])) OR (exercise therapy[Title])) OR (sports[Title])) OR (exercise tolerance[Title])) AND (CCR5[Title/Abstract])	March 5, 2024.
Pubmed 11	((((((((exercise[Title]) OR (training[Title])) OR (physical activity[Title])) OR (physical endurance[Title])) OR (exercise test[Title])) OR (exercise therapy[Title])) OR (sports[Title])) OR (exercise tolerance[Title])) AND (CXCR3[Title/Abstract])	March 5, 2024.
Pubmed 12	((((((((exercise[Title]) OR (training[Title])) OR (physical activity[Title])) OR (physical endurance[Title])) OR (exercise test[Title])) OR (exercise therapy[Title])) OR (sports[Title])) OR (exercise tolerance[Title])) AND (IL-2[Title/Abstract])	March 5, 2024.
Pubmed 13	((((((((exercise[Title]) OR (training[Title])) OR (physical activity[Title])) OR (physical endurance[Title])) OR (exercise test[Title])) OR (exercise therapy[Title])) OR (sports[Title])) OR (exercise tolerance[Title])) AND (Tumor Necrosis Factor-alpha[Title])<br><br>Filters: Humans, Adult: 19+ years; Full text, Controlled Clinical Trial, Observational Study, Randomized Controlled Trial, Humans	March 5, 2024.
Pubmed 14	((((((((exercise[Title]) OR (training[Title])) OR (physical activity[Title])) OR (physical endurance[Title])) OR (exercise test[Title])) OR (exercise therapy[Title])) OR (sports[Title])) OR (exercise tolerance[Title])) AND (TNF-α[Title])<br><br>Filters: Humans, Adult: 19+ years; Full text, Controlled Clinical Trial, Observational Study, Randomized Controlled Trial, Humans	March 5, 2024.

Pubmed 15	((((((((exercise[Title]) OR (training[Title])) OR (physical activity[Title])) OR (physical endurance[Title])) OR (exercise test[Title])) OR (exercise therapy[Title])) OR (sports[Title])) OR (exercise tolerance[Title])) AND (TNF-β[Title/Abstract])	March 5, 2024.
Pubmed 16	((((((((exercise[Title]) OR (training[Title])) OR (physical activity[Title])) OR (physical endurance[Title])) OR (exercise test[Title])) OR (exercise therapy[Title])) OR (sports[Title])) OR (exercise tolerance[Title])) AND (Tumor Necrosis Factor-beta[Title/Abstract])	March 5, 2024.
Pubmed 17	((((((((exercise[Title]) OR (training[Title])) OR (physical activity[Title])) OR (physical endurance[Title])) OR (exercise test[Title])) OR (exercise therapy[Title])) OR (sports[Title])) OR (exercise tolerance[Title])) AND (STAT1[Title/Abstract])	March 5, 2024.
Pubmed 18	((((((((exercise[Title]) OR (training[Title])) OR (physical activity[Title])) OR (physical endurance[Title])) OR (exercise test[Title])) OR (exercise therapy[Title])) OR (sports[Title])) OR (exercise tolerance[Title])) AND (STAT4[Title/Abstract])	March 5, 2024.
Pubmed 19	((((((((exercise[Title]) OR (training[Title])) OR (physical activity[Title])) OR (physical endurance[Title])) OR (exercise test[Title])) OR (exercise therapy[Title])) OR (sports[Title])) OR (exercise tolerance[Title])) AND (T-bet[Title/Abstract])	March 5, 2024.
Pubmed 20	((((((((exercise[Title]) OR (training[Title])) OR (physical activity[Title])) OR (physical endurance[Title])) OR (exercise test[Title])) OR (exercise therapy[Title])) OR (sports[Title])) OR (exercise tolerance[Title])) AND (T-bet transcription factor[Title/Abstract])	March 5, 2024.
Pubmed 21	((((((((exercise[Title]) OR (training[Title])) OR (physical activity[Title])) OR (physical endurance[Title])) OR (exercise test[Title])) OR (exercise therapy[Title])) OR (sports[Title])) OR (exercise tolerance[Title])) AND (Igg[Title/Abstract])	March 5, 2024.
Pubmed 22	((((((((exercise[Title]) OR (training[Title])) OR (physical activity[Title])) OR (physical endurance[Title])) OR (exercise test[Title])) OR (exercise therapy[Title])) OR (sports[Title])) OR (exercise tolerance[Title])) AND (Immunoglobulin G[Title/Abstract])	March 5, 2024.
Pubmed 23	((((((((exercise[Title]) OR (training[Title])) OR (physical activity[Title])) OR (physical endurance[Title])) OR (exercise test[Title])) OR (exercise therapy[Title])) OR	March 5, 2024.

	(sports[Title])) OR (exercise tolerance[Title])) AND (CCR3[Title/Abstract])	
Pubmed 24	((((((((exercise[Title]) OR (training[Title])) OR (physical activity[Title])) OR (physical endurance[Title])) OR (exercise test[Title])) OR (exercise therapy[Title])) OR (sports[Title])) OR (exercise tolerance[Title])) AND (CCR4[Title/Abstract])	March 5, 2024.
Pubmed 25	((((((((exercise[Title]) OR (training[Title])) OR (physical activity[Title])) OR (physical endurance[Title])) OR (exercise test[Title])) OR (exercise therapy[Title])) OR (sports[Title])) OR (exercise tolerance[Title])) AND (CCR8[Title/Abstract])	March 5, 2024.
Pubmed 26	((((((((exercise[Title]) OR (training[Title])) OR (physical activity[Title])) OR (physical endurance[Title])) OR (exercise test[Title])) OR (exercise therapy[Title])) OR (sports[Title])) OR (exercise tolerance[Title])) AND (CXCR4[Title/Abstract])	March 5, 2024.
Pubmed 27	((((((((exercise[Title]) OR (training[Title])) OR (physical activity[Title])) OR (physical endurance[Title])) OR (exercise test[Title])) OR (exercise therapy[Title])) OR (sports[Title])) OR (exercise tolerance[Title])) AND (IL-4[Title/Abstract])	March 5, 2024.
Pubmed 28	((((((((exercise[Title]) OR (training[Title])) OR (physical activity[Title])) OR (physical endurance[Title])) OR (exercise test[Title])) OR (exercise therapy[Title])) OR (sports[Title])) OR (exercise tolerance[Title])) AND (Interleukin-4[Title/Abstract])	March 5, 2024.
Pubmed 29	((((((((exercise[Title]) OR (training[Title])) OR (physical activity[Title])) OR (physical endurance[Title])) OR (exercise test[Title])) OR (exercise therapy[Title])) OR (sports[Title])) OR (exercise tolerance[Title])) AND (Interleukin-5[Title/Abstract])	March 5, 2024.
Pubmed 30	((((((((exercise[Title]) OR (training[Title])) OR (physical activity[Title])) OR (physical endurance[Title])) OR (exercise test[Title])) OR (exercise therapy[Title])) OR (sports[Title])) OR (exercise tolerance[Title])) AND (IL-5[Title/Abstract])	March 5, 2024.
Pubmed 31	((((((((exercise[Title]) OR (training[Title])) OR (physical activity[Title])) OR (physical endurance[Title])) OR (exercise test[Title])) OR (exercise therapy[Title])) OR (sports[Title])) OR (exercise tolerance[Title])) AND (IL-6[Title])	March 5, 2024.

	Filters: Humans, Adult: 19+ years; Full text, Controlled Clinical Trial, Observational Study, Randomized Controlled Trial, Humans	
Pubmed 32	((((((((exercise[Title]) OR (training[Title])) OR (physical activity[Title])) OR (physical endurance[Title])) OR (exercise test[Title])) OR (exercise therapy[Title])) OR (sports[Title])) OR (exercise tolerance[Title])) AND (Interleukin-6[Title])<br>Filters: Humans, Adult: 19+ years; Full text, Controlled Clinical Trial, Observational Study, Randomized Controlled Trial, Humans	March 5, 2024.
Pubmed 33	((((((((exercise[Title]) OR (training[Title])) OR (physical activity[Title])) OR (physical endurance[Title])) OR (exercise test[Title])) OR (exercise therapy[Title])) OR (sports[Title])) OR (exercise tolerance[Title])) AND (Interleukin-9[Title/Abstract])	March 5, 2024.
Pubmed 34	((((((((exercise[Title]) OR (training[Title])) OR (physical activity[Title])) OR (physical endurance[Title])) OR (exercise test[Title])) OR (exercise therapy[Title])) OR (sports[Title])) OR (exercise tolerance[Title])) AND (IL-9[Title/Abstract])	March 5, 2024.
Pubmed 35	((((((((exercise[Title]) OR (training[Title])) OR (physical activity[Title])) OR (physical endurance[Title])) OR (exercise test[Title])) OR (exercise therapy[Title])) OR (sports[Title])) OR (exercise tolerance[Title])) AND (IL-10[Title])<br><br>Filters: Humans, Adult: 19+ years; Full text, Controlled Clinical Trial, Observational Study, Randomized Controlled Trial, Humans	March 5, 2024.
Pubmed 36	((((((((exercise[Title]) OR (training[Title])) OR (physical activity[Title])) OR (physical endurance[Title])) OR (exercise test[Title])) OR (exercise therapy[Title])) OR (sports[Title])) OR (exercise tolerance[Title])) AND (Interleukin-10[Title/Abstract])	March 5, 2024.
Pubmed 37	((((((((exercise[Title]) OR (training[Title])) OR (physical activity[Title])) OR (physical endurance[Title])) OR (exercise test[Title])) OR (exercise therapy[Title])) OR (sports[Title])) OR (exercise tolerance[Title])) AND (Interleukin-13[Title/Abstract])	March 5, 2024.
Pubmed 38	((((((((exercise[Title]) OR (training[Title])) OR (physical activity[Title])) OR (physical endurance[Title])) OR (exercise test[Title])) OR (exercise therapy[Title])) OR	March 5, 2024.

	(sports[Title])) OR (exercise tolerance[Title])) AND (IL-13[Title/Abstract])	
Pubmed 39	((((((((exercise[Title]) OR (training[Title])) OR (physical activity[Title])) OR (physical endurance[Title])) OR (exercise test[Title])) OR (exercise therapy[Title])) OR (sports[Title])) OR (exercise tolerance[Title])) AND (IL-21[Title/Abstract])	March 5, 2024.
Pubmed 40	((((((((exercise[Title]) OR (training[Title])) OR (physical activity[Title])) OR (physical endurance[Title])) OR (exercise test[Title])) OR (exercise therapy[Title])) OR (sports[Title])) OR (exercise tolerance[Title])) AND (Interleukin-21[Title])	March 5, 2024.
Pubmed 41	((((((((exercise[Title]) OR (training[Title])) OR (physical activity[Title])) OR (physical endurance[Title])) OR (exercise test[Title])) OR (exercise therapy[Title])) OR (sports[Title])) OR (exercise tolerance[Title])) AND (STAT5[Title/Abstract])	March 5, 2024.
Pubmed 42	((((((((exercise[Title]) OR (training[Title])) OR (physical activity[Title])) OR (physical endurance[Title])) OR (exercise test[Title])) OR (exercise therapy[Title])) OR (sports[Title])) OR (exercise tolerance[Title])) AND (STAT6[Title/Abstract])	March 5, 2024.
Pubmed 43	((((((((exercise[Title]) OR (training[Title])) OR (physical activity[Title])) OR (physical endurance[Title])) OR (exercise test[Title])) OR (exercise therapy[Title])) OR (sports[Title])) OR (exercise tolerance[Title])) AND (GATA-3[Title/Abstract])	March 5, 2024.
Pubmed 44	((((((((exercise[Title]) OR (training[Title])) OR (physical activity[Title])) OR (physical endurance[Title])) OR (exercise test[Title])) OR (exercise therapy[Title])) OR (sports[Title])) OR (exercise tolerance[Title])) AND (IgE[Title/Abstract])	March 5, 2024.
Pubmed 45	((((((((exercise[Title]) OR (training[Title])) OR (physical activity[Title])) OR (physical endurance[Title])) OR (exercise test[Title])) OR (exercise therapy[Title])) OR (sports[Title])) OR (exercise tolerance[Title])) AND (Immunoglobulin E[Title/Abstract])	March 5, 2024.

Science Direct

https://www.sciencedirect.com/

Database	Descriptors (MESH) and Boolean algebra	Date
Science Direct 1	(Exercise OR Exercise Test OR Exercise Therapy OR Physical Endurance OR Sports OR Exercise Tolerance OR Training) AND th1 cells	March 5, 2024.
Science Direct 2	(Exercise OR Exercise Test OR Exercise Therapy OR Physical Endurance OR Sports OR Exercise Tolerance OR Training) AND th2 cells	March 5, 2024.
Science Direct 3	(Exercise OR Exercise Test OR Exercise Therapy OR Physical Endurance OR Sports OR Exercise Tolerance OR Training) AND Th1-Th2 Balance	March 5, 2024.
Science Direct 4	(Exercise OR Exercise Therapy OR Physical Endurance OR Sports OR Training) AND (th1 cells OR th2 cells OR Th1-Th2 Balance)	March 5, 2024.
Science Direct 5	(Exercise OR Exercise Test OR Exercise Therapy OR Physical Endurance OR Sports OR Exercise Tolerance OR Training) AND CD3+CD4+CD8-	March 5, 2024.
Science Direct 6	(Exercise OR Exercise Test OR Exercise Therapy OR Physical Endurance OR Sports OR Exercise Tolerance OR Training) AND IFN-gamma	March 5, 2024.
Science Direct 7	(Exercise OR Exercise Test OR Exercise Therapy OR Physical Endurance OR Sports OR Exercise Tolerance OR Training) AND Interferon-gamma	March 5, 2024.
Science Direct 8	(Exercise OR Exercise Test OR Exercise Therapy OR Physical Endurance OR Sports OR Exercise Tolerance OR Training) AND IFN-y	March 5, 2024.
Science Direct 9	(Exercise OR Exercise Test OR Exercise Therapy OR Physical Endurance OR Sports OR Exercise Tolerance OR Training) AND IL-18	March 5, 2024.
Science Direct 10	(Exercise OR Exercise Test OR Exercise Therapy OR Physical Endurance OR Sports OR Exercise Tolerance OR Training) AND CCR5	March 5, 2024.
Science Direct 11	(Exercise OR Exercise Test OR Exercise Therapy OR Physical Endurance OR Sports OR Exercise Tolerance OR Training) AND CXCR3	March 5, 2024.
Science Direct 12	(Exercise OR Exercise Test OR Exercise Therapy OR Physical Endurance OR Sports OR Exercise Tolerance OR Training) AND IL-2 AND Randomized Controlled Trial	March 5, 2024.

Science Direct 13	(Exercise OR Exercise Test OR Exercise Therapy OR Physical Endurance OR Sports OR Exercise Tolerance OR Training) AND Tumor Necrosis Factor-alpha	March 5, 2024.
Science Direct 14	(Exercise OR Exercise Test OR Exercise Therapy OR Physical Endurance OR Sports OR Exercise Tolerance OR Training) AND TNF- α AND Randomized Controlled Trial	March 5, 2024.
Science Direct 15	(Exercise OR Exercise Test OR Exercise Therapy OR Physical Endurance OR Sports OR Exercise Tolerance OR Training) AND TNF- β	March 5, 2024.
Science Direct 16	(Exercise OR Exercise Test OR Exercise Therapy OR Physical Endurance OR Sports OR Exercise Tolerance OR Training) AND Tumor Necrosis Factor-beta	March 5, 2024.
Science Direct 17	(Exercise OR Exercise Test OR Exercise Therapy OR Physical Endurance OR Sports OR Exercise Tolerance OR Training) AND STAT1	March 5, 2024.
Science Direct 18	(Exercise OR Exercise Test OR Exercise Therapy OR Physical Endurance OR Sports OR Exercise Tolerance OR Training) AND STAT4	March 5, 2024.
Science Direct 19	(Exercise OR Exercise Test OR Exercise Therapy OR Physical Endurance OR Sports OR Exercise Tolerance OR Training) AND T-bet	March 5, 2024.
Science Direct 20	(Exercise OR Exercise Test OR Exercise Therapy OR Physical Endurance OR Sports OR Exercise Tolerance OR Training) AND T-bet transcription factor	March 5, 2024.
Science Direct 21	(Exercise OR Exercise Test OR Exercise Therapy OR Physical Endurance OR Sports OR Exercise Tolerance OR Training) AND Igg	March 5, 2024.
Science Direct 22	(Exercise OR Exercise Test OR Exercise Therapy OR Physical Endurance OR Sports OR Exercise Tolerance OR Training) AND Immunoglobulin G	March 5, 2024.
Science Direct 23	(Exercise OR Exercise Test OR Exercise Therapy OR Physical Endurance OR Sports OR Exercise Tolerance OR Training) AND CCR3	March 5, 2024.
Science Direct 24	(exercise OR Exercise Test OR Exercise Therapy OR Physical Endurance OR Sports OR Exercise Tolerance OR training) AND CCR4	March 5, 2024.
Science Direct 25	(Exercise OR Exercise Test OR Exercise Therapy OR Physical Endurance OR Sports OR Exercise Tolerance OR Training) AND CCR8	March 5, 2024.

Science Direct 26	(Exercise OR Exercise Test OR Exercise Therapy OR Physical Endurance OR Sports OR Exercise Tolerance OR Training) AND CXCR4	March 5, 2024.
Science Direct 27	(Exercise OR Exercise Test OR Exercise Therapy OR Physical Endurance OR Sports OR Exercise Tolerance OR Training) AND IL-4 AND Randomized Controlled Trial	March 5, 2024.
Science Direct 28	(Exercise OR Exercise Test OR Exercise Therapy OR Physical Endurance OR Sports OR Exercise Tolerance OR Training) AND Interleukin-4	March 5, 2024.
Science Direct 29	(Exercise OR Exercise Test OR Exercise Therapy OR Physical Endurance OR Sports OR Exercise Tolerance OR Training) AND Interleukin-5	March 5, 2024.
Science Direct 30	(Exercise OR Exercise Test OR Exercise Therapy OR Physical Endurance OR Sports OR Exercise Tolerance OR Training) AND IL-5 AND Randomized Controlled Trial	March 5, 2024.
Science Direct 31	(Exercise OR Exercise Test OR Exercise Therapy OR Physical Endurance OR Sports OR Exercise Tolerance OR Training) AND IL-6 AND Randomized Controlled Trial	March 5, 2024.
Science Direct 32	(Exercise OR Exercise Test OR Exercise Therapy OR Physical Endurance OR Sports OR Exercise Tolerance OR Training) AND Interleukin-6 AND Randomized Controlled Trial	March 5, 2024.
Science Direct 33	(Exercise OR Exercise Test OR Exercise Therapy OR Physical Endurance OR Sports OR Exercise Tolerance OR Training) AND Interleukin-9	March 5, 2024.
Science Direct 34	(Exercise OR Exercise Test OR Exercise Therapy OR Physical Endurance OR Sports OR Exercise Tolerance OR Training) AND IL-9	March 5, 2024.
Science Direct 35	(Exercise OR Exercise Test OR Exercise Therapy OR Physical Endurance OR Sports OR Exercise Tolerance OR Training) AND IL-10 AND Randomized Controlled Trial	March 5, 2024.
Science Direct 36	(Exercise OR Exercise Test OR Exercise Therapy OR Physical Endurance OR Sports OR Exercise Tolerance OR Training) AND Interleukin-10	March 5, 2024.
Science Direct 37	(Exercise OR Exercise Test OR Exercise Therapy OR Physical Endurance OR Sports OR Exercise Tolerance OR Training) AND Interleukin-13	March 5, 2024.

Science Direct 38	(Exercise OR Exercise Test OR Exercise Therapy OR Physical Endurance OR Sports OR Exercise Tolerance OR Training) AND IL-13	March 5, 2024.
Science Direct 39	(Exercise OR Exercise Test OR Exercise Therapy OR Physical Endurance OR Sports OR Exercise Tolerance OR Training) AND IL-21	March 5, 2024.
Science Direct 40	(Exercise OR Exercise Test OR Exercise Therapy OR Physical Endurance OR Sports OR Exercise Tolerance OR Training) AND Interleukin-21	March 5, 2024.
Science Direct 41	(Exercise OR Exercise Test OR Exercise Therapy OR Physical Endurance OR Sports OR Exercise Tolerance OR Training) AND STAT5	March 5, 2024.
Science Direct 42	(Exercise OR Exercise Test OR Exercise Therapy OR Physical Endurance OR Sports OR Exercise Tolerance OR Training) AND STAT6	March 5, 2024.
Science Direct 43	(Exercise OR Exercise Test OR Exercise Therapy OR Physical Endurance OR Sports OR Exercise Tolerance OR Training) AND GATA-3	March 5, 2024.
Science Direct 44	(Exercise OR Exercise Test OR Exercise Therapy OR Physical Endurance OR Sports OR Exercise Tolerance OR Training) AND IgE	March 5, 2024.
Science Direct 45	(Exercise OR Exercise Test OR Exercise Therapy OR Physical Endurance OR Sports OR Exercise Tolerance OR Training) AND Immunoglobulin E	March 5, 2024.

Web of Science

http://login.webofknowledge.com/

Database	Descriptors (MESH) and Boolean algebra	Date
Web of Science 1	TS=((“Exercise” OR "Exercise Test" OR "Exercise Therapy" OR "Physical Endurance" OR “Sports” OR "Exercise Tolerance" OR “Training”) AND ("th1 cells”))	March 5, 2024.
Web of Science 2	TS=((“Exercise” OR "Exercise Test" OR "Exercise Therapy" OR "Physical Endurance" OR “Sports” OR "Exercise Tolerance" OR “Training”) AND (“th2 cells”))	March 5, 2024.
Web of Science 3	TS=((“Exercise” OR "Exercise Test" OR "Exercise Therapy" OR "Physical Endurance" OR “Sports” OR "Exercise Tolerance" OR “Training”) AND (“Th1-Th2 Balance”))	March 5, 2024.

Web of Science 4	TS=((“Exercise” OR "Exercise Test" OR "Exercise Therapy" OR "Physical Endurance" OR “Sports” OR "Exercise Tolerance" OR “Training”) AND (“th1 cells” OR “th2 cells” OR “Th1-Th2 Balance”))	March 5, 2024.
Web of Science 5	TS=((“Exercise” OR "Exercise Test" OR "Exercise Therapy" OR "Physical Endurance" OR “Sports” OR "Exercise Tolerance" OR “Training”) AND (“CD3+CD4+CD8-”))	March 5, 2024.
Web of Science 6	TS=((“Exercise” OR "Exercise Test" OR "Exercise Therapy" OR "Physical Endurance" OR “Sports” OR "Exercise Tolerance" OR “Training”) AND (“IFN-gamma”))	March 5, 2024.
Web of Science 7	TS=((“Exercise” OR "Exercise Test" OR "Exercise Therapy" OR "Physical Endurance" OR “Sports” OR "Exercise Tolerance" OR “Training”) AND (“Interferon-gamma”))	March 5, 2024.
Web of Science 8	TS=((“Exercise” OR "Exercise Test" OR "Exercise Therapy" OR "Physical Endurance" OR “Sports” OR "Exercise Tolerance" OR “Training”) AND (“IFN-y”))	March 5, 2024.
Web of Science 9	TS=((“Exercise” OR "Exercise Test" OR "Exercise Therapy" OR "Physical Endurance" OR “Sports” OR "Exercise Tolerance" OR “Training”) AND (“IL-18”))	March 5, 2024.
Web of Science 10	TS=((“Exercise” OR "Exercise Test" OR "Exercise Therapy" OR "Physical Endurance" OR “Sports” OR "Exercise Tolerance" OR “Training”) AND (“CCR5”))	March 5, 2024.
Web of Science 11	TS=((“Exercise” OR "Exercise Test" OR "Exercise Therapy" OR "Physical Endurance" OR “Sports” OR "Exercise Tolerance" OR “Training”) AND (“CXCR3”))	March 5, 2024.
Web of Science 12	TS=((“Exercise” OR "Exercise Test" OR "Exercise Therapy" OR "Physical Endurance" OR “Sports” OR "Exercise Tolerance" OR “Training”) AND (“IL-2”))	March 5, 2024.
Web of Science 13	TS=(((Exercise OR "Exercise Test" OR "Exercise Therapy" OR "Physical Endurance" OR Sports OR "Exercise Tolerance" OR Training) AND ("Tumor Necrosis Factor-alpha") AND ("Randomized Controlled Trial"))))	March 5, 2024.
Web of Science 14	TS=(((Exercise OR "Exercise Test" OR "Exercise Therapy" OR "Physical Endurance" OR Sports OR "Exercise Tolerance" OR Training) AND ("TNF-alpha") AND ("Randomized Controlled Trial")))	March 5, 2024.

Web of Science 15	TS=((“Exercise” OR "Exercise Test" OR "Exercise Therapy" OR "Physical Endurance" OR “Sports” OR "Exercise Tolerance" OR “Training”) AND (“TNF- β”))	March 5, 2024.
Web of Science 16	TS=((“Exercise” OR "Exercise Test" OR "Exercise Therapy" OR "Physical Endurance" OR “Sports” OR "Exercise Tolerance" OR “Training”) AND (“Tumor Necrosis Factor-beta”))	March 5, 2024.
Web of Science 17	TS=((“Exercise” OR "Exercise Test" OR "Exercise Therapy" OR "Physical Endurance" OR “Sports” OR "Exercise Tolerance" OR “Training”) AND (“STAT1”))	March 5, 2024.
Web of Science 18	TS=((“Exercise” OR "Exercise Test" OR "Exercise Therapy" OR "Physical Endurance" OR “Sports” OR "Exercise Tolerance" OR “Training”) AND (“STAT4”)	March 5, 2024.
Web of Science 19	TS=((“Exercise” OR "Exercise Test" OR "Exercise Therapy" OR "Physical Endurance" OR “Sports” OR "Exercise Tolerance" OR “Training”) AND (“T-bet”))	March 5, 2024.
Web of Science 20	TS=((“Exercise” OR "Exercise Test" OR "Exercise Therapy" OR "Physical Endurance" OR “Sports” OR "Exercise Tolerance" OR “Training”) AND (“T-bet transcription factor”))	March 5, 2024.
Web of Science 21	TS=((“Exercise” OR "Exercise Test" OR "Exercise Therapy" OR "Physical Endurance" OR “Sports” OR "Exercise Tolerance" OR “Training”) AND (“Igg”) AND ("Randomized Controlled Trial"))	March 5, 2024.
Web of Science 22	TS=((“Exercise” OR "Exercise Test" OR "Exercise Therapy" OR "Physical Endurance" OR “Sports” OR "Exercise Tolerance" OR “Training”) AND (“Immunoglobulin G”))	March 5, 2024.
Web of Science 23	TS=((“Exercise” OR "Exercise Test" OR "Exercise Therapy" OR "Physical Endurance" OR “Sports” OR "Exercise Tolerance" OR “Training”) AND (“CCR3”))	March 5, 2024.
Web of Science 24	TS=((“Exercise” OR "Exercise Test" OR "Exercise Therapy" OR "Physical Endurance" OR “Sports” OR "Exercise Tolerance" OR “Training”) AND (“CCR4”))	March 5, 2024.
Web of Science 25	TS=((“Exercise” OR "Exercise Test" OR "Exercise Therapy" OR "Physical Endurance" OR “Sports” OR "Exercise Tolerance" OR “Training”) AND (“CCR8”))	March 5, 2024.
Web of Science 26	TS=((“Exercise” OR "Exercise Test" OR "Exercise Therapy" OR "Physical Endurance" OR “Sports” OR "Exercise Tolerance" OR “Training”) AND (“CXCR4”))	March 5, 2024.

Web of Science 27	(TS=((("Exercise" OR "Exercise Test" OR "Exercise Therapy" OR "Physical Endurance" OR "Sports" OR "Exercise Tolerance" OR "Training") AND ("IL-4") AND ("Randomized Controlled Trial")))	March 5, 2024.
Web of Science 28	TS=((“Exercise” OR "Exercise Test" OR "Exercise Therapy" OR "Physical Endurance" OR “Sports” OR "Exercise Tolerance" OR “Training”) AND (“Interleukin-4”))	March 5, 2024.
Web of Science 29	TS=((“Exercise” OR "Exercise Test" OR "Exercise Therapy" OR "Physical Endurance" OR “Sports” OR "Exercise Tolerance" OR “Training”) AND (“Interleukin-5”))	March 5, 2024.
Web of Science 30	TS=((“Exercise” OR "Exercise Test" OR "Exercise Therapy" OR "Physical Endurance" OR “Sports” OR "Exercise Tolerance" OR “Training”) AND (“IL-5”))	March 5, 2024.
Web of Science 31	TS=(((“Exercise” OR "Exercise Test" OR "Exercise Therapy" OR "Physical Endurance" OR “Sports” OR "Exercise Tolerance" OR “Training”) AND (“IL-6”) AND ("Randomized Controlled Trial")))	March 5, 2024.
Web of Science 32	TS=(((“Exercise” OR "Exercise Test" OR "Exercise Therapy" OR "Physical Endurance" OR “Sports” OR "Exercise Tolerance" OR “Training”) AND (“Interleukin-6”) AND ("Randomized Controlled Trial")))	March 5, 2024.
Web of Science 33	TS=((“Exercise” OR "Exercise Test" OR "Exercise Therapy" OR "Physical Endurance" OR “Sports” OR "Exercise Tolerance" OR “Training”) AND (“Interleukin-9”))	March 5, 2024.
Web of Science 34	TS=((“Exercise” OR "Exercise Test" OR "Exercise Therapy" OR "Physical Endurance" OR “Sports” OR "Exercise Tolerance" OR “Training”) AND (“IL-9”))	March 5, 2024.
Web of Science 35	TS=(((“Exercise” OR "Exercise Test" OR "Exercise Therapy" OR "Physical Endurance" OR “Sports” OR "Exercise Tolerance" OR “Training”) AND (“IL-10”) AND ("Randomized Controlled Trial")))	March 5, 2024.
Web of Science 36	TS=(((“Exercise” OR "Exercise Test" OR "Exercise Therapy" OR "Physical Endurance" OR “Sports” OR "Exercise Tolerance" OR “Training”) AND (“Interleukin-10”) AND ("Randomized Controlled Trial")))	March 5, 2024.
Web of Science 37	TS=((“Exercise” OR "Exercise Test" OR "Exercise Therapy" OR "Physical Endurance" OR “Sports” OR	March 5, 2024.

	"Exercise Tolerance" OR “Training”) AND (“Interleukin-13”))	
Web of Science 38	TS=((“Exercise” OR "Exercise Test" OR "Exercise Therapy" OR "Physical Endurance" OR “Sports” OR "Exercise Tolerance" OR “Training”) AND (“IL-13”))	March 5, 2024.
Web of Science 39	TS=((“Exercise” OR "Exercise Test" OR "Exercise Therapy" OR "Physical Endurance" OR “Sports” OR "Exercise Tolerance" OR “Training”) AND (“IL-21”))	March 5, 2024.
Web of Science 40	TS=((“Exercise” OR "Exercise Test" OR "Exercise Therapy" OR "Physical Endurance" OR “Sports” OR "Exercise Tolerance" OR “Training”) AND (“Interleukin-21”))	March 5, 2024.
Web of Science 41	TS=((“Exercise” OR "Exercise Test" OR "Exercise Therapy" OR "Physical Endurance" OR “Sports” OR "Exercise Tolerance" OR “Training”) AND (“STAT5”))	March 5, 2024.
Web of Science 42	TS=((“Exercise” OR "Exercise Test" OR "Exercise Therapy" OR "Physical Endurance" OR “Sports” OR "Exercise Tolerance" OR “Training”) AND (“STAT6”))	March 5, 2024.
Web of Science 43	TS=((“Exercise” OR "Exercise Test" OR "Exercise Therapy" OR "Physical Endurance" OR “Sports” OR "Exercise Tolerance" OR “Training”) AND (“GATA-3”))	March 5, 2024.
Web of Science 44	TS=(((“Exercise” OR "Exercise Test" OR "Exercise Therapy" OR "Physical Endurance" OR “Sports” OR "Exercise Tolerance" OR “Training”) AND (“IgE”) AND ("Randomized Controlled Trial")))	March 5, 2024.
Web of Science 45	TS=((“Exercise” OR "Exercise Test" OR "Exercise Therapy" OR "Physical Endurance" OR “Sports” OR "Exercise Tolerance" OR “Training”) AND (“Immunoglobulin E”))	March 5, 2024.

Scopus

https://www.scopus.com/

Database	Descriptors (MESH) and Boolean algebra	Date
Scopus 1	TITLE-ABS-KEY ((exercise OR "Exercise Test" OR "Exercise Therapy" OR "Physical Endurance" OR sports OR "Exercise Tolerance" OR training) AND ("th1 cells") AND ("randomized controlled trial" OR "observational studies")) AND (LIMIT-TO (PUBSTAGE , "final")) AND (LIMIT-TO (DOCTYPE	March 5, 2024.

	, "ar")) AND (LIMIT-TO (LANGUAGE , "English")) AND (LIMIT-TO (EXACTKEYWORD , "Human") OR LIMIT-TO (EXACTKEYWORD , "Humans")) AND (LIMIT-TO (EXACTKEYWORD , "Adult"))	
Scopus 2	TITLE-ABS-KEY ((exercise OR "Exercise Test" OR "Exercise Therapy" OR "Physical Endurance" OR sports OR "Exercise Tolerance" OR training) AND ("th2 cells") AND ("randomized controlled trial" OR "observational studies")) AND (LIMIT-TO (PUBSTAGE , "final")) AND (LIMIT-TO (DOCTYPE , "ar")) AND (LIMIT-TO (LANGUAGE , "English")) AND (LIMIT-TO (EXACTKEYWORD , "Human") OR LIMIT-TO (EXACTKEYWORD , "Humans")) AND (LIMIT-TO (EXACTKEYWORD , "Adult"))	March 5, 2024.
Scopus 3	TITLE-ABS-KEY ((exercise OR "Exercise Test" OR "Exercise Therapy" OR "Physical Endurance" OR sports OR "Exercise Tolerance" OR training) AND ("Th1-Th2 Balance") AND ("randomized controlled trial" OR "observational studies")) AND (LIMIT-TO (PUBSTAGE , "final")) AND (LIMIT-TO (DOCTYPE , "ar")) AND (LIMIT-TO (LANGUAGE , "English")) AND (LIMIT-TO (EXACTKEYWORD , "Human") OR LIMIT-TO (EXACTKEYWORD , "Humans")) AND (LIMIT-TO (EXACTKEYWORD , "Adult"))	March 5, 2024.
Scopus 4	TITLE-ABS-KEY ((exercise OR "Exercise Test" OR "Exercise Therapy" OR "Physical Endurance" OR sports OR "Exercise Tolerance" OR training) AND (“th1 cells” OR “th2 cells” OR “Th1-Th2 Balance”) AND ("randomized controlled trial" OR "observational studies")) AND (LIMIT-TO (PUBSTAGE , "final")) AND (LIMIT-TO (DOCTYPE , "ar")) AND (LIMIT-TO (LANGUAGE , "English")) AND (LIMIT-TO (EXACTKEYWORD , "Human") OR LIMIT-TO (EXACTKEYWORD , "Humans")) AND (LIMIT-TO (EXACTKEYWORD , "Adult"))	March 5, 2024.
Scopus 5	TITLE-ABS-KEY ((exercise OR "Exercise Test" OR "Exercise Therapy" OR "Physical Endurance" OR sports OR "Exercise Tolerance" OR training) AND ("CD3+CD4+CD8-") AND ("randomized controlled trial" OR "observational studies")) AND (LIMIT-TO (PUBSTAGE , "final")) AND (LIMIT-TO (DOCTYPE , "ar")) AND (LIMIT-TO (LANGUAGE , "English")) AND (LIMIT-TO (EXACTKEYWORD , "Human") OR LIMIT-TO (EXACTKEYWORD , "Humans")) AND (LIMIT-TO (EXACTKEYWORD , "Adult"))	March 5, 2024.

Scopus 6	TITLE-ABS-KEY ((exercise OR "Exercise Test" OR "Exercise Therapy" OR "Physical Endurance" OR sports OR "Exercise Tolerance" OR training) AND ("IFN-gamma") AND ("randomized controlled trial" OR "observational studies")) AND (LIMIT-TO (PUBSTAGE , "final")) AND (LIMIT-TO (DOCTYPE , "ar")) AND (LIMIT-TO (LANGUAGE , "English")) AND (LIMIT-TO (EXACTKEYWORD , "Human") OR LIMIT-TO (EXACTKEYWORD , "Humans")) AND (LIMIT-TO (EXACTKEYWORD , "Adult"))	March 5, 2024.
Scopus 7	TITLE-ABS-KEY ((exercise OR "Exercise Test" OR "Exercise Therapy" OR "Physical Endurance" OR sports OR "Exercise Tolerance" OR training) AND ("Interferon-gamma") AND ("randomized controlled trial" OR "observational studies")) AND (LIMIT-TO (PUBSTAGE , "final")) AND (LIMIT-TO (DOCTYPE , "ar")) AND (LIMIT-TO (LANGUAGE , "English")) AND (LIMIT-TO (EXACTKEYWORD , "Human") OR LIMIT-TO (EXACTKEYWORD , "Humans")) AND (LIMIT-TO (EXACTKEYWORD , "Adult"))	March 5, 2024.
Scopus 8	TITLE-ABS-KEY ((exercise OR "Exercise Test" OR "Exercise Therapy" OR "Physical Endurance" OR sports OR "Exercise Tolerance" OR training) AND ("IFN-y") AND ("randomized controlled trial" OR "observational studies")) AND (LIMIT-TO (PUBSTAGE , "final")) AND (LIMIT-TO (DOCTYPE , "ar")) AND (LIMIT-TO (LANGUAGE , "English")) AND (LIMIT-TO (EXACTKEYWORD , "Human") OR LIMIT-TO (EXACTKEYWORD , "Humans")) AND (LIMIT-TO (EXACTKEYWORD , "Adult"))	March 5, 2024.
Scopus 9	TITLE-ABS-KEY ((exercise OR "Exercise Test" OR "Exercise Therapy" OR "Physical Endurance" OR sports OR "Exercise Tolerance" OR training) AND ("IL-18") AND ("randomized controlled trial" OR "observational studies")) AND (LIMIT-TO (PUBSTAGE , "final")) AND (LIMIT-TO (DOCTYPE , "ar")) AND (LIMIT-TO (LANGUAGE , "English")) AND (LIMIT-TO (EXACTKEYWORD , "Human") OR LIMIT-TO (EXACTKEYWORD , "Humans")) AND (LIMIT-TO (EXACTKEYWORD , "Adult"))	March 5, 2024.
Scopus 10	TITLE-ABS-KEY ((exercise OR "Exercise Test" OR "Exercise Therapy" OR "Physical Endurance" OR sports OR "Exercise Tolerance" OR training) AND ("CCR5") AND ("randomized controlled trial" OR "observational studies")) AND (LIMIT-TO (PUBSTAGE , "final")) AND (LIMIT-TO (DOCTYPE	March 5, 2024.

	, "ar")) AND (LIMIT-TO (LANGUAGE , "English")) AND (LIMIT-TO (EXACTKEYWORD , "Human") OR LIMIT-TO (EXACTKEYWORD , "Humans")) AND (LIMIT-TO (EXACTKEYWORD , "Adult"))	
Scopus 11	TITLE-ABS-KEY ((exercise OR "Exercise Test" OR "Exercise Therapy" OR "Physical Endurance" OR sports OR "Exercise Tolerance" OR training) AND ("CXCR3") AND ("randomized controlled trial" OR "observational studies")) AND (LIMIT-TO (PUBSTAGE , "final")) AND (LIMIT-TO (DOCTYPE , "ar")) AND (LIMIT-TO (LANGUAGE , "English")) AND (LIMIT-TO (EXACTKEYWORD , "Human") OR LIMIT-TO (EXACTKEYWORD , "Humans")) AND (LIMIT-TO (EXACTKEYWORD , "Adult"))	March 5, 2024.
Scopus 12	TITLE-ABS-KEY ((exercise OR "Exercise Test" OR "Exercise Therapy" OR "Physical Endurance" OR sports OR "Exercise Tolerance" OR training) AND ("IL-2") AND ("randomized controlled trial" OR "observational studies")) AND (LIMIT-TO (PUBSTAGE , "final")) AND (LIMIT-TO (DOCTYPE , "ar")) AND (LIMIT-TO (LANGUAGE , "English")) AND (LIMIT-TO (EXACTKEYWORD , "Human") OR LIMIT-TO (EXACTKEYWORD , "Humans")) AND (LIMIT-TO (EXACTKEYWORD , "Adult"))	March 5, 2024.
Scopus 13	TITLE-ABS ((exercise OR "Exercise Test" OR "Exercise Therapy" OR "Physical Endurance" OR sports OR "Exercise Tolerance" OR training) AND ("Tumor Necrosis Factor-alpha") AND ("randomized controlled trial" OR "observational studies")) AND (LIMIT-TO (PUBSTAGE , "final")) AND (LIMIT-TO (DOCTYPE , "ar")) AND (LIMIT-TO (LANGUAGE , "English")) AND (LIMIT-TO (EXACTKEYWORD , "Human") OR LIMIT-TO (EXACTKEYWORD , "Humans")) AND (LIMIT-TO (EXACTKEYWORD , "Adult"))	March 5, 2024.
Scopus 14	TITLE-ABS ((exercise OR "Exercise Test" OR "Exercise Therapy" OR "Physical Endurance" OR sports OR "Exercise Tolerance" OR training) AND ("TNF- α") AND ("randomized controlled trial" OR "observational studies")) AND (LIMIT-TO (PUBSTAGE , "final")) AND (LIMIT-TO (DOCTYPE , "ar")) AND (LIMIT-TO (LANGUAGE , "English")) AND (LIMIT-TO (EXACTKEYWORD , "Human") OR LIMIT-TO (EXACTKEYWORD , "Humans")) AND (LIMIT-TO (EXACTKEYWORD , "Adult"))	March 5, 2024.

Scopus 15	TITLE-ABS-KEY ((exercise OR "Exercise Test" OR "Exercise Therapy" OR "Physical Endurance" OR sports OR "Exercise Tolerance" OR training) AND ("TNF- β") AND ("randomized controlled trial" OR "observational studies")) AND (LIMIT-TO (PUBSTAGE , "final")) AND (LIMIT-TO (DOCTYPE , "ar")) AND (LIMIT-TO (LANGUAGE , "English")) AND (LIMIT-TO (EXACTKEYWORD , "Human") OR LIMIT-TO (EXACTKEYWORD , "Humans")) AND (LIMIT-TO (EXACTKEYWORD , "Adult"))	March 5, 2024.
Scopus 16	TITLE-ABS-KEY ((exercise OR "Exercise Test" OR "Exercise Therapy" OR "Physical Endurance" OR sports OR "Exercise Tolerance" OR training) AND ("Tumor Necrosis Factor-beta") AND ("randomized controlled trial" OR "observational studies")) AND (LIMIT-TO (PUBSTAGE , "final")) AND (LIMIT-TO (DOCTYPE , "ar")) AND (LIMIT-TO (LANGUAGE , "English")) AND (LIMIT-TO (EXACTKEYWORD , "Human") OR LIMIT-TO (EXACTKEYWORD , "Humans")) AND (LIMIT-TO (EXACTKEYWORD , "Adult"))	March 5, 2024.
Scopus 17	TITLE-ABS-KEY ((exercise OR "Exercise Test" OR "Exercise Therapy" OR "Physical Endurance" OR sports OR "Exercise Tolerance" OR training) AND ("STAT1") AND ("randomized controlled trial" OR "observational studies")) AND (LIMIT-TO (PUBSTAGE , "final")) AND (LIMIT-TO (DOCTYPE , "ar")) AND (LIMIT-TO (LANGUAGE , "English")) AND (LIMIT-TO (EXACTKEYWORD , "Human") OR LIMIT-TO (EXACTKEYWORD , "Humans")) AND (LIMIT-TO (EXACTKEYWORD , "Adult"))	March 5, 2024.
Scopus 18	TITLE-ABS-KEY ((exercise OR "Exercise Test" OR "Exercise Therapy" OR "Physical Endurance" OR sports OR "Exercise Tolerance" OR training) AND ("STAT4") AND ("randomized controlled trial" OR "observational studies")) AND (LIMIT-TO (PUBSTAGE , "final")) AND (LIMIT-TO (DOCTYPE , "ar")) AND (LIMIT-TO (LANGUAGE , "English")) AND (LIMIT-TO (EXACTKEYWORD , "Human") OR LIMIT-TO (EXACTKEYWORD , "Humans")) AND (LIMIT-TO (EXACTKEYWORD , "Adult"))	March 5, 2024.
Scopus 19	TITLE-ABS-KEY ((exercise OR "Exercise Test" OR "Exercise Therapy" OR "Physical Endurance" OR sports OR "Exercise Tolerance" OR training) AND ("T-bet") AND ("randomized controlled trial" OR "observational studies")) AND (LIMIT-TO (	March 5, 2024.

	PUBSTAGE , "final")) AND (LIMIT-TO (DOCTYPE , "ar")) AND (LIMIT-TO (LANGUAGE , "English")) AND (LIMIT-TO (EXACTKEYWORD , "Human") OR LIMIT-TO (EXACTKEYWORD , "Humans")) AND (LIMIT-TO (EXACTKEYWORD , "Adult"))	
Scopus 20	TITLE-ABS-KEY ((exercise OR "Exercise Test" OR "Exercise Therapy" OR "Physical Endurance" OR sports OR "Exercise Tolerance" OR training) AND ("T-bet transcription factor") AND ("randomized controlled trial" OR "observational studies")) AND (LIMIT-TO (PUBSTAGE , "final")) AND (LIMIT-TO (DOCTYPE , "ar")) AND (LIMIT-TO (LANGUAGE , "English")) AND (LIMIT-TO (EXACTKEYWORD , "Human") OR LIMIT-TO (EXACTKEYWORD , "Humans")) AND (LIMIT-TO (EXACTKEYWORD , "Adult"))	March 5, 2024.
Scopus 21	TITLE-ABS-KEY ((exercise OR "Exercise Test" OR "Exercise Therapy" OR "Physical Endurance" OR sports OR "Exercise Tolerance" OR training) AND ("Igg”) AND ("randomized controlled trial" OR "observational studies")) AND (LIMIT-TO (PUBSTAGE , "final")) AND (LIMIT-TO (DOCTYPE , "ar")) AND (LIMIT-TO (LANGUAGE , "English")) AND (LIMIT-TO (EXACTKEYWORD , "Human") OR LIMIT-TO (EXACTKEYWORD , "Humans")) AND (LIMIT-TO (EXACTKEYWORD , "Adult"))	March 5, 2024.
Scopus 22	TITLE-ABS-KEY ((exercise OR "Exercise Test" OR "Exercise Therapy" OR "Physical Endurance" OR sports OR "Exercise Tolerance" OR training) AND ("Immunoglobulin G") AND ("randomized controlled trial" OR "observational studies")) AND (LIMIT-TO (PUBSTAGE , "final")) AND (LIMIT-TO (DOCTYPE , "ar")) AND (LIMIT-TO (LANGUAGE , "English")) AND (LIMIT-TO (EXACTKEYWORD , "Human") OR LIMIT-TO (EXACTKEYWORD , "Humans")) AND (LIMIT-TO (EXACTKEYWORD , "Adult"))	March 5, 2024.
Scopus 23	TITLE-ABS-KEY ((exercise OR "Exercise Test" OR "Exercise Therapy" OR "Physical Endurance" OR sports OR "Exercise Tolerance" OR training) AND ("CCR3") AND ("randomized controlled trial" OR "observational studies")) AND (LIMIT-TO (PUBSTAGE , "final")) AND (LIMIT-TO (DOCTYPE , "ar")) AND (LIMIT-TO (LANGUAGE , "English")) AND (LIMIT-TO (EXACTKEYWORD , "Human") OR LIMIT-TO (EXACTKEYWORD , "Humans")) AND (LIMIT-TO (EXACTKEYWORD , "Adult"))	March 5, 2024.

Scopus 24	TITLE-ABS-KEY ((exercise OR "Exercise Test" OR "Exercise Therapy" OR "Physical Endurance" OR sports OR "Exercise Tolerance" OR training) AND ("CCR4") AND ("randomized controlled trial" OR "observational studies")) AND (LIMIT-TO (PUBSTAGE , "final")) AND (LIMIT-TO (DOCTYPE , "ar")) AND (LIMIT-TO (LANGUAGE , "English")) AND (LIMIT-TO (EXACTKEYWORD , "Human") OR LIMIT-TO (EXACTKEYWORD , "Humans")) AND (LIMIT-TO (EXACTKEYWORD , "Adult"))	March 5, 2024.
Scopus 25	TITLE-ABS-KEY ((exercise OR "Exercise Test" OR "Exercise Therapy" OR "Physical Endurance" OR sports OR "Exercise Tolerance" OR training) AND ("CCR8") AND ("randomized controlled trial" OR "observational studies")) AND (LIMIT-TO (PUBSTAGE , "final")) AND (LIMIT-TO (DOCTYPE , "ar")) AND (LIMIT-TO (LANGUAGE , "English")) AND (LIMIT-TO (EXACTKEYWORD , "Human") OR LIMIT-TO (EXACTKEYWORD , "Humans")) AND (LIMIT-TO (EXACTKEYWORD , "Adult"))	March 5, 2024.
Scopus 26	TITLE-ABS-KEY ((exercise OR "Exercise Test" OR "Exercise Therapy" OR "Physical Endurance" OR sports OR "Exercise Tolerance" OR training) AND ("CXCR4") AND ("randomized controlled trial" OR "observational studies")) AND (LIMIT-TO (PUBSTAGE , "final")) AND (LIMIT-TO (DOCTYPE , "ar")) AND (LIMIT-TO (LANGUAGE , "English")) AND (LIMIT-TO (EXACTKEYWORD , "Human") OR LIMIT-TO (EXACTKEYWORD , "Humans")) AND (LIMIT-TO (EXACTKEYWORD , "Adult"))	March 5, 2024.
Scopus 27	TITLE-ABS-KEY ((exercise OR "Exercise Test" OR "Exercise Therapy" OR "Physical Endurance" OR sports OR "Exercise Tolerance" OR training) AND ("IL-4") AND ("randomized controlled trial" OR "observational studies")) AND (LIMIT-TO (PUBSTAGE , "final")) AND (LIMIT-TO (DOCTYPE , "ar")) AND (LIMIT-TO (LANGUAGE , "English")) AND (LIMIT-TO (EXACTKEYWORD , "Human") OR LIMIT-TO (EXACTKEYWORD , "Humans")) AND (LIMIT-TO (EXACTKEYWORD , "Adult"))	March 5, 2024.
Scopus 28	TITLE-ABS-KEY ((exercise OR "Exercise Test" OR "Exercise Therapy" OR "Physical Endurance" OR sports OR "Exercise Tolerance" OR training) AND ("Interleukin-4”) AND ("randomized controlled trial" OR "observational studies")) AND (LIMIT-TO (PUBSTAGE , "final")) AND (LIMIT-TO (DOCTYPE	March 5, 2024.

	, "ar")) AND (LIMIT-TO (LANGUAGE , "English")) AND (LIMIT-TO (EXACTKEYWORD , "Human") OR LIMIT-TO (EXACTKEYWORD , "Humans")) AND (LIMIT-TO (EXACTKEYWORD , "Adult"))	
Scopus 29	TITLE-ABS-KEY ((exercise OR "Exercise Test" OR "Exercise Therapy" OR "Physical Endurance" OR sports OR "Exercise Tolerance" OR training) AND ("Interleukin-5") AND ("randomized controlled trial" OR "observational studies")) AND (LIMIT-TO (PUBSTAGE , "final")) AND (LIMIT-TO (DOCTYPE , "ar")) AND (LIMIT-TO (LANGUAGE , "English")) AND (LIMIT-TO (EXACTKEYWORD , "Human") OR LIMIT-TO (EXACTKEYWORD , "Humans")) AND (LIMIT-TO (EXACTKEYWORD , "Adult"))	March 5, 2024.
Scopus 30	TITLE-ABS-KEY ((exercise OR "Exercise Test" OR "Exercise Therapy" OR "Physical Endurance" OR sports OR "Exercise Tolerance" OR training) AND ("IL-5") AND ("randomized controlled trial" OR "observational studies")) AND (LIMIT-TO (PUBSTAGE , "final")) AND (LIMIT-TO (DOCTYPE , "ar")) AND (LIMIT-TO (LANGUAGE , "English")) AND (LIMIT-TO (EXACTKEYWORD , "Human") OR LIMIT-TO (EXACTKEYWORD , "Humans")) AND (LIMIT-TO (EXACTKEYWORD , "Adult"))	March 5, 2024.
Scopus 31	TITLE-ABS ((exercise OR "Exercise Test" OR "Exercise Therapy" OR "Physical Endurance" OR sports OR "Exercise Tolerance" OR training) AND ("IL-6") AND ("randomized controlled trial" OR "observational studies")) AND (LIMIT-TO (PUBSTAGE , "final")) AND (LIMIT-TO (DOCTYPE , "ar")) AND (LIMIT-TO (LANGUAGE , "English")) AND (LIMIT-TO (EXACTKEYWORD , "Human") OR LIMIT-TO (EXACTKEYWORD , "Humans")) AND (LIMIT-TO (EXACTKEYWORD , "Adult"))	March 5, 2024.
Scopus 32	TITLE-ABS ((exercise OR "Exercise Test" OR "Exercise Therapy" OR "Physical Endurance" OR sports OR "Exercise Tolerance" OR training) AND ("Interleukin-6") AND ("randomized controlled trial" OR "observational studies")) AND (LIMIT-TO (PUBSTAGE , "final")) AND (LIMIT-TO (DOCTYPE , "ar")) AND (LIMIT-TO (LANGUAGE , "English")) AND (LIMIT-TO (EXACTKEYWORD , "Human") OR LIMIT-TO (EXACTKEYWORD , "Humans")) AND (LIMIT-TO (EXACTKEYWORD , "Adult"))	March 5, 2024.

Scopus 33	TITLE-ABS-KEY ((exercise OR "Exercise Test" OR "Exercise Therapy" OR "Physical Endurance" OR sports OR "Exercise Tolerance" OR training) AND ("Interleukin-9") AND ("randomized controlled trial" OR "observational studies")) AND (LIMIT-TO (PUBSTAGE , "final")) AND (LIMIT-TO (DOCTYPE , "ar")) AND (LIMIT-TO (LANGUAGE , "English")) AND (LIMIT-TO (EXACTKEYWORD , "Human") OR LIMIT-TO (EXACTKEYWORD , "Humans")) AND (LIMIT-TO (EXACTKEYWORD , "Adult"))	March 5, 2024.
Scopus 34	TITLE-ABS-KEY ((exercise OR "Exercise Test" OR "Exercise Therapy" OR "Physical Endurance" OR sports OR "Exercise Tolerance" OR training) AND ("IL-9") AND ("randomized controlled trial" OR "observational studies")) AND (LIMIT-TO (PUBSTAGE , "final")) AND (LIMIT-TO (DOCTYPE , "ar")) AND (LIMIT-TO (LANGUAGE , "English")) AND (LIMIT-TO (EXACTKEYWORD , "Human") OR LIMIT-TO (EXACTKEYWORD , "Humans")) AND (LIMIT-TO (EXACTKEYWORD , "Adult"))	March 5, 2024.
Scopus 35	TITLE-ABS-KEY ((exercise OR "Exercise Test" OR "Exercise Therapy" OR "Physical Endurance" OR sports OR "Exercise Tolerance" OR training) AND ("IL-10") AND ("randomized controlled trial" OR "observational studies")) AND (LIMIT-TO (PUBSTAGE , "final")) AND (LIMIT-TO (DOCTYPE , "ar")) AND (LIMIT-TO (LANGUAGE , "English")) AND (LIMIT-TO (EXACTKEYWORD , "Human") OR LIMIT-TO (EXACTKEYWORD , "Humans")) AND (LIMIT-TO (EXACTKEYWORD , "Adult"))	March 5, 2024.
Scopus 36	TITLE-ABS-KEY ((exercise OR "Exercise Test" OR "Exercise Therapy" OR "Physical Endurance" OR sports OR "Exercise Tolerance" OR training) AND ("Interleukin-10") AND ("randomized controlled trial" OR "observational studies")) AND (LIMIT-TO (PUBSTAGE , "final")) AND (LIMIT-TO (DOCTYPE , "ar")) AND (LIMIT-TO (LANGUAGE , "English")) AND (LIMIT-TO (EXACTKEYWORD , "Human") OR LIMIT-TO (EXACTKEYWORD , "Humans")) AND (LIMIT-TO (EXACTKEYWORD , "Adult"))	March 5, 2024.
Scopus 37	TITLE-ABS-KEY ((exercise OR "Exercise Test" OR "Exercise Therapy" OR "Physical Endurance" OR sports OR "Exercise Tolerance" OR training) AND ("Interleukin-13") AND ("randomized controlled trial" OR "observational studies")) AND (LIMIT-TO (PUBSTAGE , "final")) AND (LIMIT-TO (DOCTYPE	March 5, 2024.

	, "ar")) AND (LIMIT-TO (LANGUAGE , "English")) AND (LIMIT-TO (EXACTKEYWORD , "Human") OR LIMIT-TO (EXACTKEYWORD , "Humans")) AND (LIMIT-TO (EXACTKEYWORD , "Adult"))	
Scopus 38	TITLE-ABS-KEY ((exercise OR "Exercise Test" OR "Exercise Therapy" OR "Physical Endurance" OR sports OR "Exercise Tolerance" OR training) AND ("IL-13") AND ("randomized controlled trial" OR "observational studies")) AND (LIMIT-TO (PUBSTAGE , "final")) AND (LIMIT-TO (DOCTYPE , "ar")) AND (LIMIT-TO (LANGUAGE , "English")) AND (LIMIT-TO (EXACTKEYWORD , "Human") OR LIMIT-TO (EXACTKEYWORD , "Humans")) AND (LIMIT-TO (EXACTKEYWORD , "Adult"))	March 5, 2024.
Scopus 39	TITLE-ABS-KEY ((exercise OR "Exercise Test" OR "Exercise Therapy" OR "Physical Endurance" OR sports OR "Exercise Tolerance" OR training) AND ("IL-21") AND ("randomized controlled trial" OR "observational studies")) AND (LIMIT-TO (PUBSTAGE , "final")) AND (LIMIT-TO (DOCTYPE , "ar")) AND (LIMIT-TO (LANGUAGE , "English")) AND (LIMIT-TO (EXACTKEYWORD , "Human") OR LIMIT-TO (EXACTKEYWORD , "Humans")) AND (LIMIT-TO (EXACTKEYWORD , "Adult"))	March 5, 2024.
Scopus 40	TITLE-ABS-KEY ((exercise OR "Exercise Test" OR "Exercise Therapy" OR "Physical Endurance" OR sports OR "Exercise Tolerance" OR training) AND ("Interleukin-21") AND ("randomized controlled trial" OR "observational studies")) AND (LIMIT-TO (PUBSTAGE , "final")) AND (LIMIT-TO (DOCTYPE , "ar")) AND (LIMIT-TO (LANGUAGE , "English")) AND (LIMIT-TO (EXACTKEYWORD , "Human") OR LIMIT-TO (EXACTKEYWORD , "Humans")) AND (LIMIT-TO (EXACTKEYWORD , "Adult"))	March 5, 2024.
Scopus 41	TITLE-ABS-KEY ((exercise OR "Exercise Test" OR "Exercise Therapy" OR "Physical Endurance" OR sports OR "Exercise Tolerance" OR training) AND ("STAT5") AND ("randomized controlled trial" OR "observational studies")) AND (LIMIT-TO (PUBSTAGE , "final")) AND (LIMIT-TO (DOCTYPE , "ar")) AND (LIMIT-TO (LANGUAGE , "English")) AND (LIMIT-TO (EXACTKEYWORD , "Human") OR LIMIT-TO (EXACTKEYWORD , "Humans")) AND (LIMIT-TO (EXACTKEYWORD , "Adult"))	March 5, 2024.

Scopus 42	TITLE-ABS-KEY ((exercise OR "Exercise Test" OR "Exercise Therapy" OR "Physical Endurance" OR sports OR "Exercise Tolerance" OR training) AND ("STAT6") AND ("randomized controlled trial" OR "observational studies")) AND (LIMIT-TO (PUBSTAGE , "final")) AND (LIMIT-TO (DOCTYPE , "ar")) AND (LIMIT-TO (LANGUAGE , "English")) AND (LIMIT-TO (EXACTKEYWORD , "Human") OR LIMIT-TO (EXACTKEYWORD , "Humans")) AND (LIMIT-TO (EXACTKEYWORD , "Adult"))	March 5, 2024.
Scopus 43	TITLE-ABS-KEY ((exercise OR "Exercise Test" OR "Exercise Therapy" OR "Physical Endurance" OR sports OR "Exercise Tolerance" OR training) AND ("GATA-3") AND ("randomized controlled trial" OR "observational studies")) AND (LIMIT-TO (PUBSTAGE , "final")) AND (LIMIT-TO (DOCTYPE , "ar")) AND (LIMIT-TO (LANGUAGE , "English")) AND (LIMIT-TO (EXACTKEYWORD , "Human") OR LIMIT-TO (EXACTKEYWORD , "Humans")) AND (LIMIT-TO (EXACTKEYWORD , "Adult"))	March 5, 2024.
Scopus 44	TITLE-ABS-KEY ((exercise OR "Exercise Test" OR "Exercise Therapy" OR "Physical Endurance" OR sports OR "Exercise Tolerance" OR training) AND ("IgE") AND ("randomized controlled trial" OR "observational studies")) AND (LIMIT-TO (PUBSTAGE , "final")) AND (LIMIT-TO (DOCTYPE , "ar")) AND (LIMIT-TO (LANGUAGE , "English")) AND (LIMIT-TO (EXACTKEYWORD , "Human") OR LIMIT-TO (EXACTKEYWORD , "Humans")) AND (LIMIT-TO (EXACTKEYWORD , "Adult"))	March 5, 2024.
Scopus 45	TITLE-ABS-KEY ((exercise OR "Exercise Test" OR "Exercise Therapy" OR "Physical Endurance" OR sports OR "Exercise Tolerance" OR training) AND ("Immunoglobulin E") AND ("randomized controlled trial" OR "observational studies")) AND (LIMIT-TO (PUBSTAGE , "final")) AND (LIMIT-TO (DOCTYPE , "ar")) AND (LIMIT-TO (LANGUAGE , "English")) AND (LIMIT-TO (EXACTKEYWORD , "Human") OR LIMIT-TO (EXACTKEYWORD , "Humans")) AND (LIMIT-TO (EXACTKEYWORD , "Adult"))	March 5, 2024.

Embase
https://www.embase.com

Database	Descriptors (MESH) and Boolean algebra	Date

Embase 1	(exercise:ti,ab,kw OR 'exercise test':ti,ab,kw OR 'exercise therapy':ti,ab,kw OR 'physical endurance':ti,ab,kw OR sports:ti,ab,kw OR 'exercise tolerance':ti,ab,kw OR training:ti,ab,kw) AND 'th1 cells':ti,ab,kw	March 5, 2024.
Embase 2	(exercise:ti,ab,kw OR 'exercise test':ti,ab,kw OR 'exercise therapy':ti,ab,kw OR 'physical endurance':ti,ab,kw OR sports:ti,ab,kw OR 'exercise tolerance':ti,ab,kw OR training:ti,ab,kw) AND 'th2 cells':ti,ab,kw	March 5, 2024.
Embase 3	(exercise:ti,ab,kw OR 'exercise test':ti,ab,kw OR 'exercise therapy':ti,ab,kw OR 'physical endurance':ti,ab,kw OR sports:ti,ab,kw OR 'exercise tolerance':ti,ab,kw OR training:ti,ab,kw) AND 'th1-th2 balance':ti,ab,kw	March 5, 2024.
Embase 4	((exercise:ti,ab,kw OR 'exercise test':ti,ab,kw OR 'exercise therapy':ti,ab,kw OR 'physical endurance':ti,ab,kw OR sports:ti,ab,kw OR 'exercise tolerance':ti,ab,kw OR training:ti,ab,kw) AND 'th1 cells':ti,ab,kw OR 'th2 cells':ti,ab,kw OR 'th1-th2 balance':ti,ab,kw) AND ([controlled clinical trial]/lim OR [randomized controlled trial]/lim) AND [article]/lim AND ([english]/lim OR [portuguese]/lim) AND ([adult]/lim OR [aged]/lim) AND [humans]/lim	March 5, 2024.
Embase 5	(exercise:ti,ab,kw OR 'exercise test':ti,ab,kw OR 'exercise therapy':ti,ab,kw OR 'physical endurance':ti,ab,kw OR sports:ti,ab,kw OR 'exercise tolerance':ti,ab,kw OR training:ti,ab,kw) AND cd3+cd4+cd8-:ti,ab,kw	March 5, 2024.
Embase 6	(exercise:ti,ab,kw OR 'exercise test':ti,ab,kw OR 'exercise therapy':ti,ab,kw OR 'physical endurance':ti,ab,kw OR sports:ti,ab,kw OR 'exercise tolerance':ti,ab,kw OR training:ti,ab,kw) AND 'ifn-gamma':ti,ab,kw	March 5, 2024.
Embase 7	(exercise:ti,ab,kw OR 'exercise test':ti,ab,kw OR 'exercise therapy':ti,ab,kw OR 'physical endurance':ti,ab,kw OR sports:ti,ab,kw OR 'exercise tolerance':ti,ab,kw OR training:ti,ab,kw) AND 'interferon gamma':ti,ab,kw AND ([controlled clinical trial]/lim OR [randomized controlled trial]/lim) AND [article]/lim AND ([english]/lim OR [portuguese]/lim) AND ([adult]/lim OR [aged]/lim) AND [humans]/lim	March 5, 2024.
Embase 8	(exercise:ti,ab,kw OR 'exercise test':ti,ab,kw OR 'exercise therapy':ti,ab,kw OR 'physical endurance':ti,ab,kw OR sports:ti,ab,kw OR 'exercise tolerance':ti,ab,kw OR training:ti,ab,kw) AND 'ifn y':ti,ab,kw	March 5, 2024.
Embase 9	(exercise:ti,ab,kw OR 'exercise test':ti,ab,kw OR 'exercise therapy':ti,ab,kw OR 'physical endurance':ti,ab,kw OR	March 5, 2024.

	sports:ti,ab,kw OR 'exercise tolerance':ti,ab,kw OR training:ti,ab,kw) AND 'il 18':ti,ab,kw	
Embase 10	(exercise:ti,ab,kw OR 'exercise test':ti,ab,kw OR 'exercise therapy':ti,ab,kw OR 'physical endurance':ti,ab,kw OR sports:ti,ab,kw OR 'exercise tolerance':ti,ab,kw OR training:ti,ab,kw) AND ccr5:ti,ab,kw	March 5, 2024.
Embase 11	(exercise:ti,ab,kw OR 'exercise test':ti,ab,kw OR 'exercise therapy':ti,ab,kw OR 'physical endurance':ti,ab,kw OR sports:ti,ab,kw OR 'exercise tolerance':ti,ab,kw OR training:ti,ab,kw) AND cxcr3:ti,ab,kw	March 5, 2024.
Embase 12	(exercise:ti,ab,kw OR 'exercise test':ti,ab,kw OR 'exercise therapy':ti,ab,kw OR 'physical endurance':ti,ab,kw OR sports:ti,ab,kw OR 'exercise tolerance':ti,ab,kw OR training:ti,ab,kw) AND 'il 2':ti,ab,kw AND ([controlled clinical trial]/lim OR [randomized controlled trial]/lim) AND [article]/lim AND ([english]/lim OR [portuguese]/lim) AND ([adult]/lim OR [aged]/lim) AND [humans]/lim	March 5, 2024.
Embase 13	(exercise:ti,ab,kw OR 'exercise test':ti,ab,kw OR 'exercise therapy':ti,ab,kw OR 'physical endurance':ti,ab,kw OR sports:ti,ab,kw OR 'exercise tolerance':ti,ab,kw OR training:ti,ab,kw) AND 'tumor necrosis factor-alpha':ti,ab,kw AND ([controlled clinical trial]/lim OR [randomized controlled trial]/lim) AND [article]/lim AND ([english]/lim OR [portuguese]/lim) AND ([adult]/lim OR [aged]/lim) AND [humans]/lim	March 5, 2024.
Embase 14	(exercise:ti OR 'exercise test':ti OR 'exercise therapy':ti OR 'physical endurance':ti OR sports:ti OR 'exercise tolerance':ti OR training:ti) AND 'tnf- α':ti<br><br>Usar só Título;	March 5, 2024.
Embase 15	(exercise:ti,ab,kw OR 'exercise test':ti,ab,kw OR 'exercise therapy':ti,ab,kw OR 'physical endurance':ti,ab,kw OR sports:ti,ab,kw OR 'exercise tolerance':ti,ab,kw OR training:ti,ab,kw) AND 'tnf- β':ti,ab,kw	March 5, 2024.
Embase 16	(exercise:ti,ab,kw OR 'exercise test':ti,ab,kw OR 'exercise therapy':ti,ab,kw OR 'physical endurance':ti,ab,kw OR sports:ti,ab,kw OR 'exercise tolerance':ti,ab,kw OR training:ti,ab,kw) AND 'tumor necrosis factor-beta':ti,ab,kw	March 5, 2024.
Embase 17	(exercise:ti,ab,kw OR 'exercise test':ti,ab,kw OR 'exercise therapy':ti,ab,kw OR 'physical endurance':ti,ab,kw OR	March 5, 2024.

	sports:ti,ab,kw OR 'exercise tolerance':ti,ab,kw OR training:ti,ab,kw) AND 'stat1':ti,ab,kw	
Embase 18	(exercise:ti,ab,kw OR 'exercise test':ti,ab,kw OR 'exercise therapy':ti,ab,kw OR 'physical endurance':ti,ab,kw OR sports:ti,ab,kw OR 'exercise tolerance':ti,ab,kw OR training:ti,ab,kw) AND 'stat4':ti,ab,kw	March 5, 2024.
Embase 19	(exercise:ti,ab,kw OR 'exercise test':ti,ab,kw OR 'exercise therapy':ti,ab,kw OR 'physical endurance':ti,ab,kw OR sports:ti,ab,kw OR 'exercise tolerance':ti,ab,kw OR training:ti,ab,kw) AND 't-bet':ti,ab,kw	March 5, 2024.
Embase 20	(exercise:ti,ab,kw OR 'exercise test':ti,ab,kw OR 'exercise therapy':ti,ab,kw OR 'physical endurance':ti,ab,kw OR sports:ti,ab,kw OR 'exercise tolerance':ti,ab,kw OR training:ti,ab,kw) AND 't-bet transcription factor':ti,ab,kw	March 5, 2024.
Embase 21	(exercise:ti,ab,kw OR 'exercise test':ti,ab,kw OR 'exercise therapy':ti,ab,kw OR 'physical endurance':ti,ab,kw OR sports:ti,ab,kw OR 'exercise tolerance':ti,ab,kw OR training:ti,ab,kw) AND 'igg':ti,ab,kw AND ([controlled clinical trial]/lim OR [randomized controlled trial]/lim) AND [article]/lim AND ([english]/lim OR [portuguese]/lim) AND ([adult]/lim OR [aged]/lim) AND [humans]/lim	March 5, 2024.
Embase 22	(exercise:ti,ab,kw OR 'exercise test':ti,ab,kw OR 'exercise therapy':ti,ab,kw OR 'physical endurance':ti,ab,kw OR sports:ti,ab,kw OR 'exercise tolerance':ti,ab,kw OR training:ti,ab,kw) AND 'immunoglobulin g':ti,ab,kw	March 5, 2024.
Embase 23	(exercise:ti,ab,kw OR 'exercise test':ti,ab,kw OR 'exercise therapy':ti,ab,kw OR 'physical endurance':ti,ab,kw OR sports:ti,ab,kw OR 'exercise tolerance':ti,ab,kw OR training:ti,ab,kw) AND ccr3:ti,ab,kw	March 5, 2024.
Embase 24	(exercise:ti,ab,kw OR 'exercise test':ti,ab,kw OR 'exercise therapy':ti,ab,kw OR 'physical endurance':ti,ab,kw OR sports:ti,ab,kw OR 'exercise tolerance':ti,ab,kw OR training:ti,ab,kw) AND ccr4:ti,ab,kw	March 5, 2024.
Embase 25	(exercise:ti,ab,kw OR 'exercise test':ti,ab,kw OR 'exercise therapy':ti,ab,kw OR 'physical endurance':ti,ab,kw OR sports:ti,ab,kw OR 'exercise tolerance':ti,ab,kw OR training:ti,ab,kw) AND ccr8:ti,ab,kw	March 5, 2024.
Embase 26	Exercise OR "Exercise Test" OR "Exercise Therapy" OR "Physical Endurance" OR Sports OR "Exercise Tolerance" OR Training AND CXCR4	March 5, 2024.

	(exercise:ti,ab,kw OR 'exercise test':ti,ab,kw OR 'exercise therapy':ti,ab,kw OR 'physical endurance':ti,ab,kw OR sports:ti,ab,kw OR 'exercise tolerance':ti,ab,kw OR training:ti,ab,kw) AND cxcr4:ti,ab,kw	
Embase 27	(exercise:ti,ab,kw OR 'exercise test':ti,ab,kw OR 'exercise therapy':ti,ab,kw OR 'physical endurance':ti,ab,kw OR sports:ti,ab,kw OR 'exercise tolerance':ti,ab,kw OR training:ti,ab,kw) AND 'il 4':ti,ab,kw AND ([controlled clinical trial]/lim OR [randomized controlled trial]/lim) AND [article]/lim AND ([english]/lim OR [portuguese]/lim) AND ([adult]/lim OR [aged]/lim) AND [humans]/lim	March 5, 2024.
Embase 28	(exercise:ti,ab,kw OR 'exercise test':ti,ab,kw OR 'exercise therapy':ti,ab,kw OR 'physical endurance':ti,ab,kw OR sports:ti,ab,kw OR 'exercise tolerance':ti,ab,kw OR training:ti,ab,kw) AND 'interleukin-4':ti,ab,kw	March 5, 2024.
Embase 29	(exercise:ti,ab,kw OR 'exercise test':ti,ab,kw OR 'exercise therapy':ti,ab,kw OR 'physical endurance':ti,ab,kw OR sports:ti,ab,kw OR 'exercise tolerance':ti,ab,kw OR training:ti,ab,kw) AND 'interleukin-5':ti,ab,kw	March 5, 2024.
Embase 30	(exercise:ti,ab,kw OR 'exercise test':ti,ab,kw OR 'exercise therapy':ti,ab,kw OR 'physical endurance':ti,ab,kw OR sports:ti,ab,kw OR 'exercise tolerance':ti,ab,kw OR training:ti,ab,kw) AND 'il-5':ti,ab,kw AND ([controlled clinical trial]/lim OR [randomized controlled trial]/lim) AND [article]/lim AND ([english]/lim OR [portuguese]/lim) AND ([adult]/lim OR [aged]/lim) AND [humans]/lim	March 5, 2024.
Embase 31	(exercise:ti OR 'exercise test':ti OR 'exercise therapy':ti OR 'physical endurance':ti OR sports:ti OR 'exercise tolerance':ti OR training:ti) AND 'il-6':ti AND ([controlled clinical trial]/lim OR [randomized controlled trial]/lim) AND [article]/lim AND ([english]/lim OR [portuguese]/lim) AND ([adult]/lim OR [aged]/lim) AND [humans]/lim<br><br>Usar só Título;	March 5, 2024.
Embase 32	(exercise:ti OR 'exercise test':ti OR 'exercise therapy':ti OR 'physical endurance':ti OR sports:ti OR 'exercise tolerance':ti OR training:ti) AND 'interleukin-6':ti AND ([controlled clinical trial]/lim OR [randomized controlled trial]/lim) AND [article]/lim AND ([english]/lim OR [portuguese]/lim) AND ([adult]/lim OR [aged]/lim) AND [humans]/lim	March 5, 2024.

	Usar só título;	
Embase 33	(exercise:ti,ab,kw OR 'exercise test':ti,ab,kw OR 'exercise therapy':ti,ab,kw OR 'physical endurance':ti,ab,kw OR sports:ti,ab,kw OR 'exercise tolerance':ti,ab,kw OR training:ti,ab,kw) AND 'interleukin-9':ti,ab,kw	March 5, 2024.
Embase 34	(exercise:ti,ab,kw OR 'exercise test':ti,ab,kw OR 'exercise therapy':ti,ab,kw OR 'physical endurance':ti,ab,kw OR sports:ti,ab,kw OR 'exercise tolerance':ti,ab,kw OR training:ti,ab,kw) AND 'il-9':ti,ab,kw	March 5, 2024.
Embase 35	(exercise:ti,ab,kw OR 'exercise test':ti OR 'exercise therapy':ti OR 'physical endurance':ti OR sports:ti OR 'exercise tolerance':ti OR training:ti) AND 'il-10':ti AND ([controlled clinical trial]/lim OR [randomized controlled trial]/lim) AND [article]/lim AND ([english]/lim OR [portuguese]/lim) AND ([adult]/lim OR [aged]/lim) AND [humans]/lim<br><br>Usar só título;	March 5, 2024.
Embase 36	(exercise:ti,ab,kw OR 'exercise test':ti,ab,kw OR 'exercise therapy':ti,ab,kw OR 'physical endurance':ti,ab,kw OR sports:ti,ab,kw OR 'exercise tolerance':ti,ab,kw OR training:ti,ab,kw) AND 'interleukin-10':ti,ab,kw AND ([controlled clinical trial]/lim OR [randomized controlled trial]/lim) AND [article]/lim AND ([english]/lim OR [portuguese]/lim) AND ([adult]/lim OR [aged]/lim) AND [humans]/lim	March 5, 2024.
Embase 37	(exercise:ti,ab,kw OR 'exercise test':ti,ab,kw OR 'exercise therapy':ti,ab,kw OR 'physical endurance':ti,ab,kw OR sports:ti,ab,kw OR 'exercise tolerance':ti,ab,kw OR training:ti,ab,kw) AND 'interleukin-13':ti,ab,kw	March 5, 2024.
Embase 38	(exercise:ti,ab,kw OR 'exercise test':ti,ab,kw OR 'exercise therapy':ti,ab,kw OR 'physical endurance':ti,ab,kw OR sports:ti,ab,kw OR 'exercise tolerance':ti,ab,kw OR training:ti,ab,kw) AND 'il-13':ti,ab,kw	March 5, 2024.
Embase 39	(exercise:ti,ab,kw OR 'exercise test':ti,ab,kw OR 'exercise therapy':ti,ab,kw OR 'physical endurance':ti,ab,kw OR sports:ti,ab,kw OR 'exercise tolerance':ti,ab,kw OR training:ti,ab,kw) AND 'il-21':ti,ab,kw	March 5, 2024.
Embase 40	(exercise:ti,ab,kw OR 'exercise test':ti,ab,kw OR 'exercise therapy':ti,ab,kw OR 'physical endurance':ti,ab,kw OR sports:ti,ab,kw OR 'exercise tolerance':ti,ab,kw OR training:ti,ab,kw) AND 'interleukin-21':ti,ab,kw	March 5, 2024.

Embase 41	(exercise:ti,ab,kw OR 'exercise test':ti,ab,kw OR 'exercise therapy':ti,ab,kw OR 'physical endurance':ti,ab,kw OR sports:ti,ab,kw OR 'exercise tolerance':ti,ab,kw OR training:ti,ab,kw) AND stat5:ti,ab,kw	March 5, 2024.
Embase 42	(exercise:ti,ab,kw OR 'exercise test':ti,ab,kw OR 'exercise therapy':ti,ab,kw OR 'physical endurance':ti,ab,kw OR sports:ti,ab,kw OR 'exercise tolerance':ti,ab,kw OR training:ti,ab,kw) AND stat6:ti,ab,kw	March 5, 2024.
Embase 43	(exercise:ti,ab,kw OR 'exercise test':ti,ab,kw OR 'exercise therapy':ti,ab,kw OR 'physical endurance':ti,ab,kw OR sports:ti,ab,kw OR 'exercise tolerance':ti,ab,kw OR training:ti,ab,kw) AND 'gata 3':ti,ab,kw	March 5, 2024.
Embase 44	(exercise:ti OR 'exercise test':ti OR 'exercise therapy':ti OR 'physical endurance':ti OR sports:ti OR 'exercise tolerance':ti OR training:ti) AND 'ige':ti<br><br>Usar só Título	March 5, 2024.
Embase 45	(exercise:ti,ab,kw OR 'exercise test':ti,ab,kw OR 'exercise therapy':ti,ab,kw OR 'physical endurance':ti,ab,kw OR sports:ti,ab,kw OR 'exercise tolerance':ti,ab,kw OR training:ti,ab,kw) AND 'immunoglobulin e':ti,ab,kw	March 5, 2024.
