# Supplementary material for: The effects of acute and chronic exercise on immune markers of TH1/TH2 cells in older adults: a systematic review
Source: Front Physiol. 2025 Feb 11;16:1453747. doi: 10.3389/fphys.2025.1453747 (PMC11850391; doi:10.3389/fphys.2025.1453747)
Supplement: Supplementary file 3 [file Table3.docx]

**Supplementary Table 1 -** Summary of chronic exercise effects on immunological markers in older adults.

| **Factor Evaluated** | **Positive Effect** | **No Effect** | **Negative Effect** | **Specific Comments** | **Limitations** |
| --- | --- | --- | --- | --- | --- |
| TNF-α | Abd El-Kader e Al-Jiffri, (2019), Santiago, et al. (2018), Abd El-Kader, et al. (2019), Abd El-Kader e Al-Shreef, (2018) | So, et al. (2013), Sahl, et al. (2017), Rodriguez-Miguelez, et al. (2014) | _ | Significant anti-inflammatory response after 24 weeks. | Mixing data in percentages and absolute numbers limits direct comparisons. |
| IL-6 | Abd El-Kader e Al-Jiffri, (2019), Santiago, et al. (2018), Abd El-Kader, et al. (2019), Abd El-Kader e Al-Shreef, (2018), Rodriguez-Miguelez, et al. (2014), Sbardelotto, et al. (2017) | So, et al. (2013), Forti, et al. (2016), | Sahl, et al. (2017) | Reduction observed in studies lasting ≥12 weeks. | Mixing data in percentages and absolute numbers limits the analysis. |
| IL-10 | Abd El-Kader e Al-Jiffri, (2019), Abd El-Kader, et al. (2019), Abd El-Kader e Al-Shreef, (2018), Rodriguez-Miguelez, et al. (2014) | Sbardelotto, et al. (2017) | _ | Consistent increase in moderate to high-intensity exercises. | No significant limitation for this factor. |
| CD4/CD8 | Abd El-Kader e Al-Shreef, (2018) | _ | _ | Decrease associated with improved immune balance. | Mixing data complicates interpretation of lymphocyte polarization. |
| CD3 | Abd El-Kader e Al-Shreef, (2018) | Woods, et al. (1999), Shimizu, et al. (2011) | _ | Increase observed in studies lasting ≥24 weeks. | The sample size did not allow for an appropriate analysis of gender effects. |
| CD4 | Abd El-Kader e Al-Shreef, (2018) | Woods, et al. (1999), Shimizu, et al. (2011) | _ | Increase observed in studies lasting ≥24 weeks. | The sample size did not allow for an appropriate analysis of gender effects. |
| CD8 | Abd El-Kader e Al-Shreef, (2018) | Woods, et al. (1999), Shimizu, et al. (2011) | _ | Increase observed in studies lasting ≥24 weeks. | The sample size did not allow for an appropriate analysis of gender effects. |
