# Supplementary material for: The effects of acute and chronic exercise on immune markers of TH1/TH2 cells in older adults: a systematic review
Source: Front Physiol. 2025 Feb 11;16:1453747. doi: 10.3389/fphys.2025.1453747 (PMC11850391; doi:10.3389/fphys.2025.1453747)
Supplement: Supplementary file 4 [file Table4.docx]

**Supplementary Table 2 -** Summary of acute exercise effects on immunological markers in older adults.

| **Factor Evaluated** | **Positive Effect** | **No Effect** | **Negative Effect** | **Specific Comments** | **Limitations** |
| --- | --- | --- | --- | --- | --- |
| TNF-α | _ | Windsor, et al. (2018), Minuzzi, et al. (2019) | _ | No significant changes immediately after the session. | Mixing data makes it difficult to identify clear patterns. |
| IL-6 | Minuzzi, et al. (2019) | Windsor, et al. (2018), Cornish, et al. (2018) | _ | Transient increase at maximum intensities; return to baseline within 1 hour. | Reported data in different scales (%, absolute numbers). |
| IL-10 | Minuzzi, et al. (2019) | Windsor, et al. (2018), | _ | Transient increase followed by return to baseline within 1 hour. | No significant limitation for this factor. |
| IL-4 | Minuzzi, et al. (2019) | _ | _ | Only one study reported an immediate increase. | Mixing data complicates identifying consistent trends. |
